# Delphi consensus statement on intrapartum fetal monitoring in low‐resource settings

**DOI:** 10.1002/ijgo.12724

**Published:** 2018-12-24

**Authors:** Natasha Housseine, Marieke C. Punt, Joyce L. Browne, Janneke van ‘t Hooft, Nanna Maaløe, Tarek Meguid, Gerhard B. Theron, Arie Franx, Diederick E. Grobbee, Gerard H.A. Visser, Marcus J. Rijken

**Affiliations:** ^1^ Department of Obstetrics and Gynaecology University Medical Centre Utrecht Utrecht University Utrecht Netherlands; ^2^ Julius Global Health Julius Centre for Health Sciences and Primary Care University Medical Centre Utrecht Utrecht University Utrecht Netherlands; ^3^ Department of Obstetrics and Gynaecology Mnazi Mmoja Hospital Zanzibar Tanzania; ^4^ Department of Obstetrics and Gynaecology Academic Medical Center Amsterdam Netherlands; ^5^ Global Health Section Department of Public Health University of Copenhagen Copenhagen Denmark; ^6^ School of Health and Medical Science State University of Zanzibar Zanzibar Tanzania; ^7^ Department of Obstetrics and Gynecology Faculty of Medicine and Health Sciences Stellenbosch University Cape Town South Africa; ^8^ FIGO Committee Safe Motherhood and Newborn Health FIGO London UK

**Keywords:** Admission test, Fetal monitoring, Guidelines, Intermittent auscultation, Low‐ and middle‐income countries, Low‐resource settings

## Abstract

**Objective:**

To determine acceptable and achievable strategies of intrapartum fetal monitoring in busy low‐resource settings.

**Methods:**

Three rounds of online Delphi surveys were conducted between January 1 and October 31, 2017. International experts with experience in low‐resource settings scored the importance of intrapartum fetal monitoring methods.

**Results:**

71 experts completed all three rounds (28 midwives, 43 obstetricians). Consensus was reached on (1) need for an admission test, (2) handheld Doppler for intrapartum fetal monitoring, (3) intermittent auscultation (IA) every 30 minutes for low‐risk pregnancies during the first stage of labor and after every contraction for high‐risk pregnancies in the second stage, (4) contraction monitoring hourly for low‐risk pregnancies in the first stage, and (5) adjunctive tests. Consensus was not reached on frequency of IA or contraction monitoring for high‐risk women in the first stage or low‐risk women in the second stage of labor.

**Conclusion:**

There is a gap between international recommendations and what is physically possible in many labor wards in low‐resource settings. Research on how to effectively implement the consensus on fetal assessment at admission and use of handheld Doppler during labor and delivery is crucial to support staff in achieving the best possible care in low‐resource settings.

## Abstract 2

International fetal monitoring strategies are not achievable in many low‐resource settings. Consensus was reached that admission assessment and Doppler are the first steps towards acceptable surveillance.

## 1 INTRODUCTION

Almost all perinatal deaths occur in low‐ and middle‐income countries (LMICs), and half occur intrapartum.^1^ The daily reality of many low‐resource health facilities impedes timely and high‐quality labor care.^2^, ^3^ On admission, women have unknown or insufficiently known risk status owing to inadequate prenatal care and suboptimal assessment at first contact with a skilled birth attendant.^4^, ^5^ During labor, women receive suboptimal support, including poor monitoring of their babies, who may die unnoticed.^2^, ^6^ Fetal monitoring may provide crucial information on the adequacy of fetal oxygenation during labor for timely and appropriate management.^7^

Evidence is lacking to develop an ideal intrapartum fetal monitoring system to improve perinatal outcomes. Practice is guided by expert consensus and obstetric culture, which often originate in high‐income countries.^8^ For low‐risk pregnancies, assessment of fetal heart rate (FHR) by intermittent auscultation (IA) for 30–60 seconds is commonly recommended every 15 or 30 minutes in the active phase of the first stage of labor, and after every contraction or at 5‐minute intervals in the second stage. The strength and frequency of contractions are generally determined every 30 minutes over a 10‐minute period. For high‐risk pregnancies, continuous cardiotocography (CTG) is recommended (Table 1).

**Table 1:** Fetal and contraction monitoring recommendations in renowned international and national guidelines

Guideline^a^	Pregnancy risk status	Intermittent auscultation				Contractions	
		Frequency during first stage	Frequency during second stage	Timing	Duration	Timing	Duration
1. FIGO, 2015	Low	15 min	Every 5 min	During and at least 30 s after contraction	At least 60 s	Before and during FHR auscultation, in order to detect at least two contractions	10 min
	High	Continuous EFM					
2. WHO IMPAC, 2000	Low	Every 30 min	Every 5 min	After contraction	1 min	Every 30 min	10 min
	High	No recommendation					
3. NICE, 2014, UK	Low	At least every 15 min	At least every 5 min	After contraction	At least 1 min	Half hourly	Not stated
	High	Continuous EFM					
4. RANZCOG, 2014, Australia/New Zealand	Low	Every 15–30 min	After contraction or at least every 5 min	Commence toward end of contraction, and continue for at least 30–60 s after contraction		Not stated	Not stated
	High	Continuous EFM					
5. ACNM, 2010, USA	Low	Every 15–30 min	Every 5–15 min	After contraction	30–60 s	Not stated	Not stated
	High	Continuous EFM					
6. ACOG, 2009, USA	Low	At least every 15 min	At least every 5 min	Not stated	Not stated	Not stated	Not stated
	High	Continuous EFM					
7. SOGC, 2007, Canada	Low	15–30 min	5 min	After contraction	30–60 s	Not stated	Not stated
	High	Continuous EFM					
8. RCOG, 2001 UK	Low	At least every 15 min	At least every 5 min	After contraction	At least 60 s	Not stated	Not stated
	High	Continuous EFM					

Abbreviation: EFM, electronic fetal monitoring; FHR, fetal heart rate.

a References to the guidelines are given in Supplementary Table S5.

A substantial mismatch exists between international guidelines and what is locally achievable. In high‐volume low‐resource settings, the ratio of skilled birth attendants (SBAs) to deliveries often exceeds one to three.^9^, ^10^ The challenges in labor monitoring are well known, yet current recommendations do not take into consideration the limited (human) resources in settings where one‐to‐one care and/or CTG are not feasible. Poor performance may result from an overwhelming workload and demotivation caused by unrealistic expectations.^11^

Feasible implementation strategies are needed to support overwhelmed SBAs and help them to manage the high number of deliveries. Evidence indicates that clinical recommendations that are realistic, simple, and easy to understand have a greater chance of translation into practice.^12^ WHO encourages regional, national, and subnational adaptation of their guidelines.^13^ It is therefore paramount to explore how international guidelines can be adapted to more closely reflect the reality at the targeted maternity units that need the most guidance.

With use of a Delphi procedure, we aimed to determine a package of achievable strategies of intrapartum fetal monitoring for busy low‐resource maternity wards with a focus on admission tests, FHR monitoring, adjunctive tests, and contraction monitoring in relation to low‐ and high‐risk pregnancies in the first and second stage of labor.

## 2 MATERIALS AND METHODS

The online Delphi study was conducted among nurses/midwives, obstetricians, and pediatricians in accordance with predefined objectives, criteria for expert panel selection, and statistical methods. Three Delphi rounds took place between January 1 and October 31, 2017. The Core Outcome Set‐Standards for Reporting was used.^14^ The ethics board of the University Medical Center Utrecht (reference, WAG/nt/16/033902) decided that no formal ethical approval was required.

**Figure 1:**
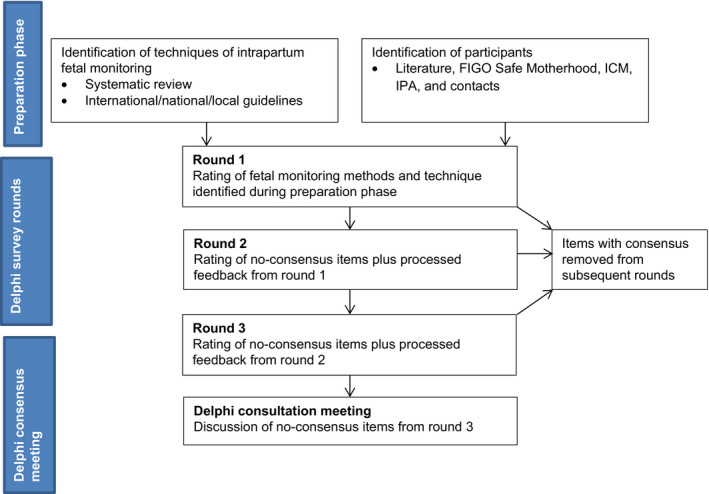
Flowchart of the consensus procedure. Abbreviations: FIGO, International Federation of Gynecology and Obstetrics; ICM, International Confederation of Midwives; IPA, International Pediatric Association.

A project steering committee was established to coordinate the different phases of the project and consisted of four obstetricians (GHAV, GBT, TM and MJR), one epidemiologist (JB), and two methodologists (JH and NM) with experience in consensus methods (Fig. 1).

A systematic review was conducted to identify the different fetal monitoring techniques available in LMICs (registered in PROSPERO: CRD42016038679). Five electronic databases (Pubmed/MEDLINE, Cochrane Library, EMBASE, POPLINE, and Global Health Library) were searched to identify studies with a title or abstract containing MeSH/Emtree terms related to “intrapartum,” “fetal surveillance,” “outcomes,” and “low‐ and middle‐income countries.” In addition, international, national, and local guidelines were searched for recommendations on the frequency and duration of FHR and contraction monitoring (Table 1).^9^ The definitions used to guide participants are given in Box 1, and the outcomes are listed in Supplementary Tables [Link], [Link].

Box 1 **Definitions of terms provided to participants during the Delphi rounds.**

1

**Table:** 

Low‐resource setting	Busy maternity ward with limited (human) resources, where birth attendants have many women in labor to take care of at the same time
Low maternal risk pregnancy	Uncomplicated prenatal history (with no previous cesarean section)
Low fetal risk pregnancy	Based on the expert's clinical judgment, which may include a favorable admission test
High fetal risk pregnancy	Based on the expert's clinical judgment, which may include an unfavorable admission test or intrapartum events, for example, oxytocin usage or meconium
Low‐risk pregnancy	Uncomplicated prenatal history (with no previous cesarean) and low fetal risk pregnancy
Admission test	Admission test here means ANY one or more fetal assessments performed when a pregnant woman in labor is admitted to the maternity unit. It may include physical examination or bedside tests
First stage of active phase of labor	Cervical dilatation from 4 to 10 cm
Second stage of active phase of labor	Fully dilated and pushing
Adjunctive test	Supplemental/additional test next to fetal heart rate monitoring for confirming fetal wellbeing
Amniotic fluid index assessment	Estimation of the amount of amniotic fluid by ultrasound
Fetal acoustic stimulation test	Detection of fetal response to sound stimulation by a vibroacoustic stimulator
Fetal pulse oximetry	Monitoring of the fetal hemoglobin oxygen saturation
Fetal scalp stimulation test	Assessing fetal response to stroking of the fetal scalp via vagina
Rapid biophysical profile	Ultrasound detection of fetal movement and amniotic fluid index
Biophysical profile	Nonstress by cardiotocography and ultrasound assessment of: fetal movement, fetal tone, fetal breathing, and amniotic fluid volume
Umbilical artery Doppler assessment	Doppler ultrasonography of the umbilical arteries
Normal/reassuring fetal heart rate by intermittent auscultation	Fetal heart rate between 110 and 160 bpm
Suboptimal/non‐reassuring fetal heart rate by intermittent auscultation	A baseline fetal heart rate of 100–109 or 161–180 bpm
Abnormal fetal heart rate by intermittent auscultation	A baseline fetal heart rate <100 or >180 bpm or repetitive or prolonged decelerations >3 min
Intrauterine resuscitation	Measures aimed at increasing oxygen delivery to the fetus; e.g., stopping oxytocin, maternal repositioning, and administration of intravenous fluids and oxygen

A three‐round electronic Delphi survey is a well‐established consensus method allowing anonymous consultation with controlled feedback.^15^ The invited stakeholder groups included midwives, obstetricians, and pediatricians (i.e., neonatologists or pediatricians involved in neonatal care) with work experience in low‐resource settings, and/or experts in fetal monitoring.

To identify suitable experts, international professional organizations were contacted by email and asked to forward the invitation to its members. Additional experts were identified through published clinical research of relevance. A formal email invitation was sent to all experts identified. The Delphi survey was developed in SurveyMonkey and pilot‐tested by members of the project steering committee with subsequent adjustments. Three rounds were conducted, each with an average closing date of 4 weeks. Stakeholders who did not participate in a round were not invited for subsequent rounds.

The stakeholders were asked to make recommendations that were minimal, safe, and achievable for birth attendants in low‐resource settings (i.e., busy maternity units with limited [human] resources, where one SBA provides care for multiple laboring women simultaneously). Throughout the rounds, options were presented for the following areas of fetal monitoring: admission tests, low‐ and high‐risk pregnancies, first stage of active phase and second stage of labor, suboptimal and abnormal FHR, adjunctive tests, and contraction monitoring.

Stakeholders were asked to quantify their level of support for potential recommendations via a 5‐item Likert scale (1, strongly disagree; 2, disagree; 3, neutral; 4, agree; 5, strongly agree) and to answer additional multiple‐choice questions. Space was provided for free‐text feedback.

Survey responses were analyzed with SPSS version 23.0 (IBM, Armonk, NY, USA). For each outcome, frequencies and percentages of level of agreement were calculated per stakeholder group. In subsequent rounds, the individual and stakeholder group results were relayed back. For each round, all comments were analyzed and suggestions were added to subsequent rounds (i.e., clarifications, rewording of sentences, or additional definitions).

Consensus was defined a priori as at least 70% of stakeholders scoring an item as “agree/strongly agree” and less than 15% scoring it as “disagree/strongly disagree.” Exclusion of items required at least 70% of stakeholders scoring the item as “disagree/strongly disagree” and less than 15% scoring it as “agree/strongly agree.” Items that did not meet these criteria were classified as “no consensus.” For multiple‐choice questions, a level of 70% agreement was used. If consensus was reached, participants were informed and the outcome was left out from subsequent rounds. Outcomes that nearly reached consensus were discussed by the steering committee for a final decision. Attrition analysis was performed by comparing the medians of outcomes among those who participated in subsequent rounds to the medians of those who did not.

After completion of the three Delphi rounds, an expert consultation meeting was held in The Netherlands with an online dial‐in option (January 4, 2018). All participants who completed the third round were invited. In total, seven obstetricians, six midwives, and all members of the steering committee attended. The final results of the Delphi rounds were discussed, but no further attempt to reach consensus was taken. After each round, including the consultation meeting, open coding was performed on all free‐text comments by highlighting and constructing themes, and the text was summarized.

## 3 RESULTS

In total, 215 experts were invited to participate in the Delphi survey, consisting of 83 (38.6%) midwives, 92 (42.8%) obstetricians, and 40 (18.6%) pediatricians; 51 (23.7%), 82 (38.1%), and 82 (38.1%) experts originated from low‐, middle‐, and high‐income countries, respectively. In the first round, 107 (49.8%) responded; in the second round, 84 (79%) responded; and in the third round, 71 (90%) responded (Table 2).

**Table 2:** Characteristics of the participants by round.^a^

Characteristic	Round 1 **(**n = 215)	Round 2 **(**n = 107)	Round 3 **(**n = 79)
No. of respondents	107 (50)	84 (79)	71 (90)
Profession			
Nurse/midwife	48 (45)	35 (42)	28 (39)
Obstetrician	49 (46)	44 (52)	43 (61)
Pediatrician	10 (10)	5 (6)	0 (0)
Experience in low/middle income country			
Yes	94 (88)	72 (86)	59 (83)
<1 y	8 (7)	8 (10)	5 (7)
1–5 y	27 (25)	21 (25)	17 (24)
6–10 y	23 (21)	14 (17)	12 (17)
>10 y	36 (34)	29 (35)	25 (35)
Sex			
Male	43 (40)	35 (42)	29 (41)
Female	64 (60)	49 (58)	42 (59)
Age, y			
25–35	16 (15)	12 (14)	8 (11)
36–45	29 (27)	23 (27)	20 (28)
46–59	34 (32)	25 (30)	21 (30)
≥60	28 (28)	24 (29)	22 (31)
No. of countries of origin^b^	39	35	30
Low income	10 (9)	7 (8)	5 (7)
Middle income	36 (34)	27 (32)	22 (31)
High income	61 (57)	50 (60)	44 (62)
Involved in patient care	84 (79)	66 (79)	57 (80)
Involved in research	55 (51.4)	48 (57)	42 (59)
Involved in teaching/training	76 (71)	57 (68)	48 (68)
Involved in guideline developments	78 (73)	63 (75)	53 (75)

a Values are given as number (percentage) or number.

b Participating countries: Afghanistan, Australia, Bangladesh, Barbados, Belgium, Brazil, Canada, Colombia, Denmark, Eritrea, Ethiopia, France, Germany, Indonesia, Italy, Kenya, Lesotho, Malawi, Malaysia, Namibia, Netherlands, New Zealand, Nigeria, Norway, Philippines, Portugal, Russia, South Africa, Sri Lanka, Suriname, Sweden, Tanzania, The Gambia, Tunisia, The United Kingdom, Uruguay, United States of America, Venezuela, and Zambia.

The respondents, of whom 83%–88% had experience in low‐resource settings (90% for more than 1 year), originated from 39 different countries. 13 participants without experience in low‐resource settings were recommended by the consultation organizations on the basis of their expertise in fetal monitoring. Because only five pediatricians responded in the second round, this group was deemed too small to reach meaningful consensus in subsequent rounds and was therefore not invited to the third round.

A summary of outcomes is shown in Table 3, and all details on agreement are given in Supplementary Tables [Link], [Link]. Feedback given during the rounds and consultation meeting related to clarification of definitions, additional outcomes to consider, achievability of recommendation, lack of evidence, and need for implementation research to inform clinical context‐specific practice in low‐resource settings (Supplementary Table S3). Attrition analysis showed similar scores between rounds (Supplementary Table S4).

**Table 3:** Summary of consensus for the various outcomes (i.e. in/out/no consensus)

Minimum set of intrapartum assessments (round of consensus)	Outcomes not included (round of exclusion if consensus reached)
Admission tests	
Fetal movement by maternal perception (round 2)	CTG
Gestational age (round 1)	Amniotic fluid index
Fundal height (round 1)	Fetal acoustic stimulation test
Maternal blood loss (round 1)	Fetal scalp stimulation test (round 3)
Intermittent auscultation by: handheld Doppler (round 1) or Pinard stethoscope (round 2)	Fetal movement by ultrasound detection
Meconium‐staining of amniotic fluid (round 3)	Fetal pulse oximetry (round 3)
	Rapid biophysical profile (round 3)
	Umbilical artery Doppler velocity (round 3)
Fetal heart rate monitoring	
Low‐risk pregnancies in 1st stage of active phase of labor	
Method: handheld Doppler (round 1) or Pinard (round 2)	CTG: non‐invasive
Frequency: every 30 min (round 3)	CTG: invasive (round 1)
	Duration of IA
Low‐risk pregnancies in 2nd stage of labor	
Method: handheld Doppler (round 1) or Pinard (round 2)	CTG: non‐invasive
	CTG: invasive (round 1)
	Frequency of IA
	Duration of IA
High‐risk pregnancies in 1st stage of active phase of labor	
Method: Doppler (round 1) or Pinard^a^ (round 3)	CTG: non‐invasive
Duration: 60 seconds (round 2)	CTG: invasive (round 3)
	Frequency of IA
High‐risk pregnancies in 2nd stage of labor	
Method: Doppler (round 1), non‐invasive CTG^b^ or Pinard^c^ (round 3)	CTG invasive (round 3)
Frequency: After every contraction (round 2)	
Duration: 60 s (round 2)	
Suboptimal FHR	
Frequency in 2nd stage: After every contraction (round 2)	Frequency of IA in 1st stage of active phase of labor
Abnormal FHR	Within how many minutes should fetal heart be confirmed?
Adjunctive tests	
Normal FHR	Normal/suboptimal/abnormal FHR in 1st and 2nd stage of labor^d^
Meconium (round 3)	Amniotic fluid index
Maternal wellbeing (round 2)	Fetal acoustic stimulation test
None: No additional test, continue monitoring FHR (round 2)	Fetal scalp stimulation test
Suboptimal FHR (in 1st stage of active phase and 2nd stage of labor)	Fetal movement by maternal perception
Meconium (rounds 1 and 3), intrauterine resuscitation (rounds 2 and 3)	Fetal movement by ultrasound detection
Abnormal FHR	Fetal pulse oximetry
1st stage of labor: meconium, intrauterine resuscitation (round 2)	Rapid biophysical profile
	Umbilical artery Doppler velocity
2nd stage of labor: intrauterine resuscitation, no additional test, immediate delivery (round 3)	None: No additional test, continue monitoring FHR
	None: No additional test, immediate delivery
Contraction monitoring	
Low‐risk pregnancies in 1st stage of active phase of labor	
Frequency: hourly (round 3)	
Duration: 10 minutes (round 3)	
High‐risk pregnancies in 1st stage of active phase of labor	
Duration: 10 min (round 3)	Frequency

Abbreviations: CTG: cardiotocography; FHR, fetal heart rate monitoring; IA, intermittent auscultation.

a Agree: midwives:77.8%, obstetricians:73.2%. Disagree: midwives:11.1%, obstetricians:19.5%.

b Agree: midwives:69.2%, obstetricians:73.8%. Disagree: midwives:11.5%, obstetricians:14.3%.

c Agree: midwives:73.1%, obstetricians:78.6%. Disagree: midwives:15.4%, obstetricians:14.3%.

d See Table S1 and S2 for consensus out/ no consensus according to FHR and stage of labor.

Participants strongly favored an admission test for all women who present in labor (midwives, 27/28 [96%]; obstetricians, 41/42 [98%]), consisting of history taking and physical examination including IA. Intermittent auscultation by handheld Doppler was widely recommended for both low‐ and high‐risk pregnancies in the first and second stage of labor, whereas Pinard stethoscope was considered primarily acceptable for low‐risk pregnancies.

For low‐risk pregnancies in the first stage (active phase) of labor, the frequency of IA should be every 30 minutes. For high‐risk pregnancies in the second stage, including those with suboptimal/non‐reassuring FHR, use of handheld Doppler was favored after every contraction. Participants also recommended the use of continuous CTG for high‐risk pregnancies in the second stage (18 [69%] of the 26 midwives who completed this item; obstetricians, 31/42 [74%]). The recommended duration of IA for high‐risk pregnancies was at least 60 seconds. For low‐risk pregnancies in the first stage of labor, consensus was reached that contractions should be checked for 10 minutes at least every hour. In the case of ruptured membranes, meconium‐stained liquor could be used as an adjunctive test for fetal wellbeing, irrespective of FHR. Similarly, the fetal reaction to intrauterine resuscitation (defined in Box 1) should be considered in the case of a suboptimal or abnormal FHR. If FHR is abnormal in the second stage, immediate delivery should be expedited rather than further monitoring.

No consensus was reached on the frequency of monitoring for (1) high‐risk pregnancies (17/26 [65%] midwives and 31/42 [74%] obstetricians suggested every 15 minutes); (2) suboptimal FHR in the first stage (17/26 [65%] midwives and 33/42 [79%] obstetricians suggested after every contraction); and (3) low‐risk pregnancies in the second stage (19/26 [73%] and 5/26 [19%] midwives, and 21/42 [50%] and 15/42 [36%] obstetricians suggested after every contraction or every 5 minutes, respectively). No consensus was reached on the duration of IA in low‐risk pregnancies. There was also no agreement on the frequency of contraction monitoring in high‐risk pregnancies (20/26 [77%] midwives suggested every 30 minutes for 10 minutes; 26/42 [62%] obstetricians suggested every hour for 10 minutes).

There was no consensus on monitoring after an abnormal FHR is detected in the active phase of labor. However, the majority of participants thought that fetal compromise should be confirmed within 5 minutes with a decision of whether or not to expedite immediate delivery (midwives,18/26 [69%]; obstetricians, 23/42 [55%]).

Fetal acoustic stimulation and scalp stimulation tests (defined in Box 1) were mostly excluded as forms of fetal monitoring on admission to the labor ward or as adjunctive tests. Midwives strongly favored, but obstetricians opposed, the use of maternal perception of fetal movement in the intrapartum period (agreement in the first stage for normal, suboptimal, and abnormal FHR was, respectively, 23/26 [89%], 21/26 [81%], and 22/26 [85%] for midwives versus 23/42 [55%], 16/42 [38%], and 5/42 [12%] for obstetricians) (Supplementary Table S1).

## 4 DISCUSSION

The international Delphi procedure with input from experts from 39 countries resulted in consensus on five aspects of intrapartum fetal monitoring for busy low‐resource maternity units: (1) need for an admission test, (2) handheld Doppler as the recommended method of intrapartum FHR monitoring, (3) frequency of IA for low‐risk pregnancies during the first stage of labor and frequency of IA for high‐risk women in the second stage, (4) frequency of contraction monitoring for low‐risk pregnancies in the first stage of labor, (5) adjunctive tests to FHR monitoring. There was no consensus on the frequency of FHR or contraction monitoring for high‐risk women in the first stage of labor, nor for low‐risk pregnancies in the second stage. There was disagreement between midwives and obstetricians on the use of adjunctive tests, maternal perception of fetal movements, and fetal stimulation. Feedback from participants suggested two main reasons for disagreement: lack of evidence to guide expert opinion, and no single definition of “busy low‐resource setting.”

The study involved a substantial group of participants (n=107) representing 39 countries. Importantly, the majority (>80%) of experts had experience of labor care in low‐resource settings. The subsequent attrition of particularly midwives and pediatricians is, however, a limitation. These two stakeholder groups were not represented in the steering committee, and the effect of this cannot be ruled out. Although effort was taken to include a proportionate representation of experts from LMIC, the response rates of these experts were lower than those in high‐income countries, possibly owing to access to the online survey. An inherent limitation is linked to the expert‐based approach, which was chosen because of the lack of scientific evidence. However, the results may provide a foundation for future studies to generate evidence. Variation in the experts’ definitions of pregnancy risk status, low‐resource setting, and suboptimal/abnormal FHR, as well as their preferred methods in their clinical practice, might also have influenced responses.

In the present study, fetal assessment on admission and monitoring during the second stage of labor were identified as key intrapartum points for perinatal survival, enabling triage and expedited instrumental vaginal delivery, respectively.^16^ A rapid, low‐cost, low‐technology triage algorithm based on the findings of risk assessment and physical examination—for example, an adapted version of the Intelligent Structured Intermittent Auscultation framework 17—to triage laboring women into appropriate levels of fetal monitoring in low‐resource settings might support the implementation of these recommendations. Furthermore, IA on admission is a simple quality‐of‐care indicator to evaluate and improve intra‐hospital care.^2^, ^18^

Although a Pinard stethoscope was considered acceptable, handheld Doppler was seen as the preferred method for FHR monitoring. The Pinard stethoscope is easily available in all settings and requires no consumables, such as batteries or gel. Users, however, might struggle to hear FHR in busy and noisy wards. The effect of handheld Doppler on operative delivery rates is not well established, and the instrument may not be readily available in low‐resource settings owing to the associated consumables and associated costs.^19^ Innovations are being developed to overcome such problems.^20^

Cardiotocography was considered useful only for high‐risk pregnancies during the second stage. This contrasts with the international guidelines summarized in Table 1, which all advice continuous CTG monitoring for high‐risk women during the whole period of labor (apart from the WHO, which does not express an opinion on this matter). In the present survey, however, concerns were raised about the validity and feasibility of CTG, even for high‐risk women in the second stage, owing to a lack of evidence of improvement in perinatal outcomes and increased rates of cesarean in high‐income countries, high costs and maintenance, regular training of staff, and difficulties in the interpretation of CTG traces.^8^, ^21^ Nonetheless, this consensus reveals the underlying urgent need for optimal FHR surveillance and timely management (e.g., instrumental deliveries) in the second stage, which may prevent stillbirth or severe birth asphyxia.^22^ Meeting this need calls for novel FHR monitoring innovations as an alternative to CTG, such as the Moyo monitor (Laerdal Global Health, Stavanger, Norway) for intermittent prolonged monitoring of FHR.^23^

In the absence of evidence on optimal and minimal safe frequencies and duration of IA and monitoring of contractions, there was little deviation from established guidelines, except for the recommended hourly monitoring of contractions. A key methodologic finding of the study may be how clearly difficult it is for experts to deviate from international guidelines or common practice (culture and tradition) in order to reach reality. Yet, the actuality of the gap in human resources in many labor wards in LMICs implies that such guidelines are physically unachievable.^6^, ^9^, ^24^ If one SBA simultaneously attends three laboring women with FHR according to the recommended 30‐minutes interval, there would be no time for any other activities. Respectful patient care during labor requires high frequency and sufficient communication about fetal monitoring. Until human resource needs are met and rigorous evidence is available, respectful guidance for overworked health providers requires an achievable frequency of assessments for routine intrapartum care.^9^ Therefore, it should be explored how future Delphi studies can better include the “reality‐based evidence,” including task prioritization, in the decision‐making for best possible management in resource‐constrained settings.

Invasive adjunctive tests were discouraged because of concerns of improper procedures and interpretation, higher risk of infection, and sustainability. For an abnormal FHR, a change in maternal position and use of affordable tocolytic drugs (if available) to stop or reduce contractions were considered important, particularly in the case of a long decision‐to‐delivery interval. Non‐invasive alternative adjunctive tests, including maternal perception of fetal movements, and fetal acoustic and scalp stimulation tests, received little support from the experts. Strikingly, obstetricians opposed the use of maternal perception of fetal movement during labor. Likely reasons are its apparent absence in actual clinical practice and limited evidence.^25^ During the consultation meeting, however, it was suggested that the presence of fetal movement helps to confirm fetal wellbeing and might aid in guiding clinical management, a point that was agreed among midwives.

In conclusion, consensus was reached that intrapartum fetal monitoring in low‐resource settings might benefit from a standard admission test and the use of IA by handheld Doppler in both stages of labor. With regard to the study's consensus on FHR assessment frequencies, reality proves them to be unachievable in many high‐volume maternity units in low‐income countries. This emphasizes the unacceptable reality and calls for more and well‐trained staff. Implementation research on how to strengthen admission assessment and intrapartum surveillance, and related effects on perinatal survival is paramount. Consideration should be given to clinical experience, patient preference, and locally derived data for developing achievable context‐specific guidelines toward reducing intrapartum morbidity and mortality in low‐resource settings.

## AUTHOR CONTRIBUTIONS

NH, MP, JB, and MJR conceived and designed the Delphi Survey with contributions from JVH, NM, TM, GBT, GHAV, DEG, and AF. NH carried out data acquisition and analysis. NH interpreted the results with substantial contributions from GHAV, MJR, and DEG. NH drafted the manuscript; MP, JB, JVH, NM, TM, GBT, GHAV, DEG, AF, and MJR revised the manuscript. All authors reviewed, approved, and agreed to be accountable for the final manuscript.

## CONFLICTS OF INTEREST

The authors have no conflicts of interest.

## Supporting information


**Table S1.** Results per stakeholder group and per round (Likert scale).

**Table S2.** Results per stakeholder group and per round (multiple choice questions).

**Table S3.** Feedback from participants of Delphi rounds and/or consultation meeting.

**Table S4.** Attrition analysis.

**Table S5.** References for guidelines listed in Table 1.
