# Supplementary material for: Delphi consensus statement on intrapartum fetal monitoring in low‐resource settings
Source: Int J Gynaecol Obstet. 2018 Dec 24;146(1):8–16. doi: 10.1002/ijgo.12724 (PMC7379246; doi:10.1002/ijgo.12724)
Supplement: Supplementary file 1 — Table S1. Results per stakeholder group and per round (Likert scale). [file IJGO-146-8-s001.docx]

| **Table S1: Results per stakeholder group and per round (Likert scale)** | | | | | | | | | | | | | | | | | | | | | | | | | | | | | |  |
| --- | --- | --- | --- | --- | --- | --- | --- | --- | --- | --- | --- | --- | --- | --- | --- | --- | --- | --- | --- | --- | --- | --- | --- | --- | --- | --- | --- | --- | --- | --- |
|  | ROUND 1 | | | | | | | | | | | ROUND 2 | | | | | | | | | | | ROUND 3 | | | | | | Consensus  (IN/OUT/NO) |  |
|  | Agree | | | | Neutral | | | | Disagree | | | Agree | | | | Neutral | | | | Disagree | | | Agree | | Neutral | | Disagree | |  |  |
|  | M | O | P | M | | O | P | M | | O | P | M | O | P | M | | O | P | M | | O | P | M | O | M | O | M | O |  |  |
| Would you be in favour of an admission test to assess foetal well-being? | 80.4 (37/46) | 83.3 (40/48 | 80 (8/10) | 4.3 (2/46) | | 2.1 (1/48) | 10.0 (1/10) | 15.2 (7/46) | | 14.6 (7/48) | 10.0 (1/10) |  | | | | | | | | | | | 96.4 (27/28) | 97.6 (41/42) | 3.6 (1/28) | 0.0  (0/42) | 0.0  (0/28) | 2.4 (1/42) | IN |  |
| If you are to use an admission test for pregnant woman in labour in a low resource setting, how do you rate EACH of the following for the assessment of foetal wellbeing on admission to the labour ward in a low resource setting? | | | | | | | | | | | | | | | | | | | | | | | | | | | | |  |  |
| Cardiotocogram (CTG) | 54.5 (24/44) | 46.7 (21/45) | 66.7 (6/9) | 11.4 (5/44) | | 8.9 (21/45) | 0.0 (0/9) | 34.1 (15/44) | | 44.4 (20/45 | 33.3 (3/9) | 32.4 (12/37) | 25.6 (12/43) | 0.0 (0/4) | 18.9 (7/37) | | 11.6 (5/43) | 50.0 (2/4) | 48.6 (18/37) | | 60.5 (26/43) | 50.0 (2/4) | 34.6 (9/26) | 14.3 (6/42) | 11.5 (3/26) | 7.1 (3/42) | 53.8 14/26) | 78.6 (33/42) | NO |  |
| Foetal heart rate auscultation by Pinard/DeLee stethoscope | 92.9 (39/42) | 80.4 (37/46) | 66.7 (6/9) | 2.4 (1/42) | | 80.4 (37/46) | 66.7 (6/9) | 4.8 (2/42) | | 10.9 (5/37) | 0.0  (0/9) | 91.9 (34/37) | 81.4 (35/43) | 75.0 (3/4) | 0.0 (0/37) | | 9.3 (4/43) | 25.0 (1/4) | 8.1  (3/37) | | 9.3 (4/43) | 0.0 (0/4) |  | | | | | | IN |  |
| Foetal heart rate auscultation by hand-held Doppler | 81.8 (36/44) | 85.4 (41/48) | 77.8 (7/9) | 11.4 (5/44) | | 4.2 (2/41) | 22.2 (7/9) | 6.8 (3/44) | | 10.4 (5/48) | 0.0 (0/9) |  | | | | | | | | | | | | | | | | | IN |  |
| Amniotic Fluid Index/assessment | 37.5 (15/40) | 17.8 (8/45) | 11.1 (1/9) | 22.5 (9/40) | | 28.9 (13/45) | 44.4 (4/9) | 40.0 (16/40) | | 53.3 (24/45) | 44.4 (4/9) | 16.2 (6/37) | 4.7 (2/43) | 0.0 (0/4) | 27.0 (10/37) | | 20.9 (9/43) | 50.0 (2/4) | 56.8 (21/37) | | 74.4 (32/43) | 50.0 (2/4) | 15.4  (4/26) | 7.1  (3/42) | 15.4 (4/26) | 7.1 (3/42) | 69.2 (18/26) | 85.7 (36/42) | NO |  |
| Foetal Acoustic Stimulation Test | 22.0 (9/41) | 14.0 (6/43) | 12.5 (1/8) | 39.0 (16/41) | | 25.6 (11/43) | 37.5 (3/8) | 39.0 (16/41) | | 60.5 (26/43) | 50.0 (4/8) | 18.9 (7/37) | 7.0 (3/43) | 25.0 (1/4) | 29.7 (11/37) | | 16.3 (7/43) | 75.0 (3/4) | 51.4 (19/37) | | 76.7 (33/43) | 0 .0  (0/4) | 19.2  (5/26) | 11.9 (5/42) | 15.4 (4/26) | 2.4 (1/42) | 65.4 (17/26) | 85.7 (36/42 | NO |  |
| Foetal Movement Assessment by maternal perception | 73.2 (30/41) | 71.7 (33/46) | 100.0 (9/9) | 14.6 (6/41) | | 6.5 (3/46) | 0.0 (0/9) | 12.2 (5/41) | | 21.7 (10/46) | 0 (0/9) | 91.9 (34/37) | 79.1 (34/43) | 100.0 (4/4) | 5.4 (2/37) | | 14.0 (6/43) | 0.0 (0/4) | 2.7  (1/37) | | 7.0 (3/43) | 0.0 (0/4) |  | | | | | | IN |  |
| Foetal Movement Assessment by ultrasound detection | 32.5  (13/40) | 13.6  (6/44) | 55.6  (5/9) | 25.0(  10/40) | | 9.1  (4/44) | 22.2  (2/9) | 42.5  (17/40) | | 77.3  (34/44) | 22.2  (2/9) | 32.4  (12/37) | 2.3  (1/43) | 25.0  (1/4) | 16.2  (6/37) | | 14.0  (6/43) | 50.0  (2/4) | 51.4  (19/37) | | 83.7  (36/43) | 25.0  (1/4) | 19.2  (5/26) | 4.8  (2/42) | 11.5  (3/26) | 2.4  (1/42) | 69.2  (18/26) | 92.9  (39/42) | OUT |  |
| Rapid Biophysical profile | 22.5  (9/40) | 15.6  (7/45) | 22.2  (2/9) | 27.5  (11/40) | | 13.3  (6/45) | 33.3  (3/9) | 50.0  (20/40) | | 71.1  (32/45) | 44.4  (4/9) | 27.0  (10/37) | 0.0  (0/43) | 0.0  (0/4) | 24.3  (9/37) | | 7.0  (3/43) | 25.0  (1/4) | 48.6  (18/37) | | 93.0  (40/43) | 75.0  (3/4) | 11.5  (3/26) | 7.1  (3/42) | 15.4  (4/26) | 2.4  (1/42) | 73.1  (19/26) | 90.5  (38/42) | OUT** |  |
| Umbilical Artery Doppler assessment | 20.0 (8/40) | 4.4 (2/45) | 22.2 (2/9) | 27.5 (11/40) | | 11.1 (5/45) | 11.1 (1/9) | 52.5 (21/40) | | 84.4 (38/45) | 66.7 (6/9) | 18.9 (7/37) | 0.0 (0/43) | 0.0 (0/4) | 16.2 (6/37) | | 0.0 (0/43) | 0.0 (0/4) | 64.9 (24/37) | | 100.0 (43/43) | 100.0 (4/4) | 15.4  (4/26) | 2.4 (1/42) | 11.5 (3/26) | 0.0 (0/42) | 73.1 (19/26) | 97.6 (41/42) | OUT** |  |
| Foetal Pulse Oximetry | 25.0 (10/40) | 4.8 (2/42) | 0.0 (0/9) | 20.0 (8/40) | | 7.1 (3/42) | 66.7 (6/9) | 55.0 (22/40) | | 88.1 (37/42) | 33.3 (3/9) | 18.9 (7/37) | 0.0 (0/43) | 0.0 (0/4) | 18.9 (7/37) | | 0.0 (0/43) | 0.0 (0/4) | 62.2 (24/37) | | 100.0 (43/43) | 100.0 (4/4) | 15.4  (4/26) | 2.4 (1/42) | 7.7 (2/26) | 0 (0/42) | 76.9 (20/26) | 97.6 (41/42) | OUT** |  |
| Assessment of meconium stained liquor | 84.1 (37/44) | 61.7 (29/47) | 66.7 (6/9) | 9.1 (4/44) | | 17.0 (8/47) | 22.2 (2/9) | 6.8 (3/44) | | 21.0 (10/47) | 11.1 (1/9) | 86.5 (32/37) | 67.4 (29/43) | 75.0 (3/4) | 5.4 (2/37) | | 11.6 (5/43) | 0.0 (0/4) | 8.1  (3/37) | | 20.9 (9/43) | 25.0 (1/4) | 88.5 (23/26) | 92.9 (39/42) | 7.7 (2/26) | 2.4 (1/42) | 3.8 (1/26) | 4.8 (2/42) | IN |  |
| Assessment of gestational age | 83.7 (36/43) | 82.6 (38/46) | 75.0 (6/8) | 9.3 (4/43) | | 8.7 (4/46) | 25.0 (2/8) | 7.0 (3/43) | | 8.7 (4/46) | 0.0 (0/8) |  | | | | | | | | | | | | | | | | | IN |  |
| Assessment of fundal height | 88.4 (38/43) | 85.1 (40/47) | 88.9 (8/9) | 4.7 (2/43) | | 4.3 (2/47) | 0.0  (0/9) | 7.0 (3/43) | | 10.6 (5/47) | 11.1 (1/9) |  | | | | | | | | | | | | | | | | | IN |  |
| Assessment of maternal blood loss | 85.4 (35/41) | 67.4 (31/46) | 88.9 (8/9) | 9.8 (4/41) | | 19.6 (9/46) | 0.0  (0/9) | 4.9 (2/41) | | 13.0 (6/46) | 11.1 (1/9) |  | | | | | | | | | | | | | | | | | IN |  |
| Foetal Scalp Stimulation Test* |  | | | | | | | | | | | 13.5 (5/37) | 16.3 (7/43) | 0.0  (0/4) | 40.5 (15/37) | | 32.6 (14/43) | 75.0 (3/4) | 45.9 (17/37) | | 51.2 (22/43) | 25.0 (1/4) | 3.8 (1/26) | 11.9 (5/42) | 19.2 (5/26) | 11.9 (5/42) | 76.9 (20/26) | 76.2 (32/42) | OUT |  |
| What would be the appropriate method for foetal heart rate monitoring  for low maternal and low foetal risk pregnancies in the first stage of active phase of labour? | | | | | | | | | | | | | | | | | | | | | | | | | | | | |  |  |
| Cardiotocogram (CTG) - Non-invasive (abdominal) | 41.7 (15/36) | 18.2 (8/44) | 37.5 (3/8) | 11.1 (4/36) | | 11.4 (5/44) | 25.0 (2/8) | 47.2 (17/36) | | 70.5 (31/44) | 37.5 (3/8) | 30.6 (11/36) | 7.1 (3/42) | 0.0  (0/4) | 2.8 (1/36) | | 14.3 (6/42) | 25.0 (1/4) | 66.7 (24/36) | | 78.6 (33/42) | 75.0 (3/4) | 17.4 (4/23) | 0.0 (0/40) | 17.4 (4/23) | 2.5 (1/40) | 65.2 (15/23) | 97.5 (39/40) | NO |  |
| Cardiotocogram (CTG) - Invasive (foetal scalp electrodes) | 5.6 (2/36) | 0.0 (0/44) | 0.0 (0/8) | 22.2 (8/36) | | 2.3 (1/44) | 12.5 (1/8) | 72.2 (26/36) | | 97.7 (43/44) | 87.5 (7/8) |  | | | | | | | | | | | | | | | | | OUT |  |
| Intermittent Auscultation – Pinard/ DeLee stethoscope | 90.0 (36/40) | 87.0 (40/46) | 62.5 (5/8) | 5.0 (2/40) | | 10.9 (5/46) | 25.0 (2/8) | 5.0 (2/40) | | 2.2 (1/46) | 12.5 (1/8) | 94.4 (34/36) | 76.2 (32/42) | 100.0 (4/4) | 2.8 (1/36) | | 14.3 (6/42) | 0.0 (0/4) | 2.8  (1/36) | | 9.5 (4/42) | 0.0 (0/4) |  | | | | | | IN |  |
| Intermittent Auscultation - Hand-held Doppler | 77.3 (34/44) | 93.6 (44/47) | 75.0 (6/8) | 11.4 (5/44) | | 6.4 (3/47) | 12.5 (1/8) | 11.4 (5/44) | | 0.0 (0/47) | 12.5 (1/8) |  | | | | | | | | | | | | | | | | | IN |  |
| In a low resource setting, what would be your method for foetal heart rate monitoring for low maternal and low foetal in the second stage of active phase of labour? | | | | | | | | | | | | | | | | | | | | | | | | | | | | |  |  |
| Cardiotocogram (CTG) - Non-invasive | 43.2 (16/37) | 25.6 (11/43) | 50.0 (4/8) | 16.2 (6/37) | | 11.6 (5/43) | 12.5 (1/8) | 40.5 (15/37) | | 62.8 (27/43) | 37.5 (3/8) | 30.6 (11/36) | 16.7 (7/42) | 25.0 (1/4) | 25.0 (9/36) | | 4.8 (2/42) | 0.0 (0/4) | 44.4 (16/36) | | 78.6 (33/42) | 75 (3/4) | 34.8 (8/23) | 5.0 (2/40) | 0.0 (0/23) | 7.5 (3/40) | 65.2 (15/23) | 87.5 (35/40) | NO |  |
| Cardiotocogram (CTG) - Invasive | 13.9 (5/36) | 0.0 (0/41) | 0.0 (0/8) | 11.1 (4/36) | | 4.9 (2/41) | 0.0 (0/8) | 75.0 (27/36) | | 95.1 (39/41) | 100.0 (8/8) |  | | | | | | | | | | | | | | | | | OUT |  |
| Intermittent Auscultation – Pinard/DeLee stethoscope | 87.5 (35/40) | 78.3 (36/46) | 25.0 (2/8) | 5.0 (2/40) | | 15.2 (7/46) | 50.0 (4/8) | 7.5 (3/40) | | 6.5 (3/46) | 25.0 (2/8) | 86.1 (31/36) | 78.6 (33/42) | 100 (4/4) | 5.6 (2/36) | | 9.5 (4/42) | 0.0  (0/4) | 8.3  (3/36) | | 11.9 (5/42) | 0.0 (0/4) |  | | | | | | IN |  |
| Intermittent Auscultation - Hand-held Doppler | 80.5 (33/41) | 91.5 (43/47) | 75.0 (6/8) | 7.3 (3/41) | | 8.5 (4/47) | 25.0 (2/8) | 12.2 (5/41) | | 0.0  (0/47) | 0.0  (0/8) |  | | | | | | | | | | |  | | | | | | IN |  |
| In a low resource setting, what would be your method for foetal monitoring in low maternal, high foetal risk pregnancies in the first stage of active phase of labour? | | | | | | | | | | | | | | | | | | | | | | | | | | | | |  |  |
| Cardiotocogram (CTG) - Non-invasive | 81.6 (31/38) | 72.3 (34/47) | 50.0 (4/8) | 10.5 (4/38) | | 8.5 (4/47) | 25 (2/8) | 7.9 (3/38) | | 19.1 (9/47) | 25 (2/8) | 75.0 (27/36) | 82.5 (33/40) | 82.5 (3/4) | 8.3 (3/36 | | 7.5 (3/40) | 0.0 (0/4) | 16.7 (6/36) | | 10.0 (4/40) | 25.0 (1/4) | 65.4 (17/26) | 78.6 (33/42) | 19.2 (5/26) | 7.1 (3/42) | 15.4 (4/26) | 14.3 (6/42) | NO |  |
| Cardiotocogram (CTG) - Invasive | 30.6 (11/36) | 20.0 (8/40) | 0.0 (0/8) | 30.6 (11/36) | | 10.0 (4/40) | 25.0 (2/8) | 38.9 (14/36) | | 70.0 (28/40) | 75.0 (6/8) | 11.1 (4/36) | 7.5 (3/40) | 0.0  (0/4) | 30.5 (11/36) | | 15.0 (6/40) | 25.0 (1/4) | 58.3  (21/36) | | 77.5  (31/40) | 75.0 (3/4) | 11.5 (3/26) | 9.5 (4/42) | 19.2 (5/26) | 19.0 (8/42) | 69.2 (18/26 | 71.4 (30/42) | OUT** |  |
| Intermittent Auscultation – Pinard/ DeLee stethoscope | 76.3 (29/38) | 60.5 (26/43) | 37.5 (3/8) | 10.5 (4/38) | | 14.0 (6/43) | 37.5 (3/8) | 13.2 (5/38) | | 25.6 (11/43) | 25.0 (2/8) | 83.3 (30/36) | 52.5 (21/40) | 100.0 (4/4) | 2.8  (1/36) | | 20.0 (8/40) | 0.0 (0/4) | 13.8  (5/36) | | 27.5  (11/40) | 0.0 (0/4) | 76.9 (20/26) | 73.8 (31/42) | 11.5 (3/26) | 7.1 (3/42) | 11.5 (3/26) | 19.0 (8/42) | IN** |  |
| Intermittent Auscultation - Hand-held Doppler | 75.0 (27/36) | 81.8 (36/44) | 71.4 (5/7) | 16.7 (6/36) | | 9.1 (4/44) | 14.3 (1/7) | 8.3 (3/36) | | 9.1 (4/44) | 14.3 (1/7) |  | | | | | | | | | | |  | | | | | | IN |  |
| In a low resource setting, what would be your method for foetal monitoring in low maternal, high foetal risk pregnancies in the second stage of active phase of labour? | | | | | | | | | | | | | | | | | | | | | | | | | | | | |  |  |
| Cardiotocogram (CTG) - Non-invasive | 75 (27/36) | 73.9 (34/46) | 62.5 (5/8) | 13.9 (5/36) | | 4.3 (2/46) | 12.5 (1/8) | 11.1 (4/36) | | 21.7 (10/46) | 25.0 (2/8) | 77.1 (27/35) | 69.2 (27/39) | 75.0  (3/4) | 8.6  (3/35) | | 10.3 (4/39) | 0.0 (0/4) | 14.3 (5/35) | | 20.5 (8/39) | 25.0  (1/4) | 69.2 (18/26) | 73.8 (31/42) | 19.2 (5/26) | 11.9 (5/42) | 11.5 (3/26) | 14.3 (6/42) | IN** |  |
| Cardiotocogram (CTG) - Invasive | 37.1 (13/35) | 34.1 (14/41) | 0.0 (0/6) | 22.9 (8/35) | | 9.8 (4/41) | 33.3 (2/6) | 40.0 (14/35) | | 56.1 (23/41) | 66.7 (4/6) | 22.9 (8/35) | 20.5 (8/39) | 0.0 (0/4) | 14.3  (5/35) | | 7.7  (3/39) | 25.0  (1/4) | 62.9  (22/35) | | 71.8 (28/39) | 75.0 (3/4) | 11.5 (3/26) | 9.5 (4/42) | 15.4 (4/26) | 16.7 (7/42) | 73.1 (19/26) | 73.8 (31/42) | OUT |  |
| Intermittent Auscultation - Pinard/ DeLee stethoscope | 68.4 (26/38) | 60.5 (26/43) | 50.0 (4/8) | 18.4 (7/38) | | 11.6 (5/43) | 12.5 (1/8) | 13.2 (5/38) | | 27.9 (12/43) | 37.5 (3/8) | 82.8 (29/35) | 56.4 (22/39) | 75.0 (3/4) | 5.7 (2/35) | | 17.9  (7/39) | 25.0  (1/4) | 11.4  (4/35) | | 25.6 (10/39) | 0.0 (0/4) | 73.1 (19/25) | 78.6 (33/42) | 11.5 (3/26) | 7.1 (3/42) | 15.4 (4/26) | 14.3 (5/42) | IN** |  |
| Intermittent Auscultation - Hand-held Doppler | 83.8 (31/37) | 75.0 (33/44) | 87.5 (7/8) | 8.1 (3/37) | | 11.4 (5/44) | 0.0 (0/8) | 8.1 (3/37) | | 13.6 (6/44) | 12.5 (1/8) |  | | | | | | | | | | | | | | | | | IN |  |
| When foetal heart rate is normal, which supplemental test would you like for monitoring foetal well-being? | | | | | | | | | | | | | | | | | | | | | | | | | | | | |  |  |
| Foetal Scalp Sampling | 9.4 (3/32) | 0 .0 (0/41) | 12.5 (1/8) | 3.1 (1/32) | | 2.4 (1/41) | 12.5 (1/8) | 87.5 (28/32) | | 97.6 (40/41) | 75.0 (6/8) |  | | | | | | | | | | | | | | | | | OUT |  |
| Foetal Pulse Oximetry | 12.1 (4/33) | 0.0 (0/40) | 0.0 (0/8) | 12.1 (4/33) | | 5.0 (2/40) | 12.5 (1/8) | 75.8 (25/33) | | 95.0 (38/40) | 87.5 (7/8) |  | | | | | | | | | | | | | | | | | OUT |  |
| Foetal Scalp Stimulation Test | 6.3 (2/32) | 2.6 (1/39) | 0.0 (0/8) | 21.9 (7/32) | | 7.7 (3/39) | 12.5 (1/8) | 71.9 (23/32) | | 89.7 (35/39) | 87.5 (7/8) |  | | | | | | | | | | | | | | | | | OUT |  |
| Assessment of liquor for meconium | 74.4 (29/39) | 59.5 (25/42) | 62.5 (5/8) | 2.6 (1/39) | | 11.9 (5/42) | 37.5 (3/8) | 23.1 (9/39) | | 28.6 (12/42) | 0 .0 (0/8) | 82.9 (29/35) | 66.7 (26/39) | 75.0 (3/4) | 14.3 (5/35) | | 7.7  (3/39) | 0.0 (0/4) | 33.3  (1/3) | | 25.6 (10/39) | 25.0 (1/4) | 96.2 (25/26) | 88.1 (37/42) | 3.8 (1/26) | 2.4 (1/42) | 0.0 (0/26) | 9.5 (4/42) | IN |  |
| Assessment of foetal movements by maternal perception | 84.6 (33/39) | 34.1 (15/44) | 87.5 (7/8) | 5.1 (2/39) | | 20.5 (9/44) | 12.5 (1/8) | 10.3 (4/39) | | 45.5 (20/44) | 0.0 (0/8) | 85.7 (30/35) | 43.6 (17/39) | 100.0 (4/4) | 14.3 (5/35) | | 15.4 (6/39) | 0.0 (0/4) | 0.0 (0/35) | | 41.0 (16/39) | 0.0 (0/4) | 88.5 (23/26) | 54.8 (23/42) | 11.5 (3/26) | 14.3 (6/42) | 0.0 (0/26) | 31.0 (13/42) | NO |  |
| Assessment of foetal movement by ultrasound detection | 26.5 (9/34) | 0.0 (0/41) | 12.5 (1/8) | 23.5 (8/34) | | 2.4 (1/41) | 50.0 (4/8) | 50.0 (17/34) | | 97.6 (40/41) | 37.5 (3/8) | 17.1 (6/35) | 5.1 (2/39) | 50.0 (2/4) | 14.3 (5/35) | | 7.7 (3/39) | 0.0 (0/4) | 68.6 (24/35) | | 87.2 (34/39) | 50.0 (2/4) | 3.8  (1/26) | 2.4 (1/42) | 23.1 (6/26) | 0.0 (0/42) | 73.1 (19/26) | 97.6 (41/42) | OUT |  |
| Foetal Acoustic Stimulation Test | 12.5 (4/32) | 10.0 (4/40) | 0.0 (0/8) | 31.3 (10/32) | | 12.5 (5/40) | 37.5 (3/8) | 56.3 (18/32) | | 77.5 (31/40) | 62.5 (5/8) | 14.3 (5/35) | 10.3 (4/39) | 0.0 (0/4) | 22.8 (8/35) | | 5.1 (2/39) | 0.0 (0/4) | 62.9 (22/35) | | 84.6 (33/39) | 100.0 (4/4) | 7.7  (2/26) | 11.9 (5/42) | 15.4 (4/26) | 2.4 (1/42) | 76.9 (20/26) | 85.7 (36/42) | OUT |  |
| None: No additional test, continue monitoring | 60.6 (20/33) | 67.5 (27/40) | 14.3 (1/7) | 18.2 (6/33) | | 20.0 (8/40) | 42.9 (3/7) | 21.2 (7/33) | | 12.5 (5/40) | 42.9 (3/7) | 71.4 (25/35) | 79.5 (31/39) | 50.0 (2/4) | 5.7 (2/35) | | 7.7 (3/39) | 25.0 (1/4) | 22.9 (8/35) | | 12.8 (5/39) | 25.0 (1/4) |  | | | | | | IN |  |
| Monitor maternal wellbeing* |  | | | | | | | | | | | 91.4 (32/35) | 87.2 (34/39) | 100.0 (4/4) | 5.7 (2/35) | | 7.7 (3/39) | 0.0 (0/4) | 2.9 (1/35) | | 5.1 (2/39) | 0.0 (0/4) |  | | | | | | IN |  |
| In case foetal heart beat is suboptimal, what adjunctive test (s) would you do to confirm foetal well-being in the first stage of active phase of labour? | | | | | | | | | | | | | | | | | | | | | | | | | | | | | |  |
| Foetal Scalp Sampling | 48.6 (17/35) | 29.3 (12/41) | 12.5 (1/8) | 20.0 (7/35) | | 19.5 (8/41) | 25.0 (2/8) | 31.4 (11/35) | | 51.2 (21/41) | 62.5 (5/8) | 22.9 (8/35) | 33.3 (13/39) | 25.0 (1/4) | 37.1 (13/35) | | 15.4 (6/39) | 0.0 (0/4) | 40.0 (14/35) | | 51.3 (20/39) | 75.0 (3/4) | 3.8  (1/26) | 21.4 (9/42) | 38.5 (10/26) | 9.5 (4/42) | 57.7 (15/26) | 69.0 (29/42) | NO |  |
| Foetal Pulse Oximetry | 27.3 (9/33) | 12.5 (5/40) | 25.0 (2/8) | 33.3 (11/33) | | 17.5 (7/40) | 12.5 (1/8) | 39.4 (13/33) | | 70.0 (28/40) | 62.5 (5/8) | 25.7 (9/35) | 7.7 (3/39) | 0.0 (0/4) | 22.8 (8/35) | | 12.8 (5/39) | 0.0 (0/4) | 51.4 (18/35) | | 79.5 (31/39) | 100.0 (4/4) | 11.5 (3/26) | 2.4 (1/42) | 15.4 (4/26) | 4.8 (2/42) | 73.1 (19/26) | 92.9 (39/42) | OUT |  |
| Foetal Scalp Stimulation Test | 25.8 (8/31) | 55.8 (24/43) | 0.0 (0/8) | 29.0 (9/31) | | 7.0 (3/43) | 50.0 (4/8) | 45.2 (14/31) | | 37.2 (16/43) | 50.0 (4/8) | 34.3 (12/35) | 61.5 (24/39) | 0.0 (0/4) | 28.6 (10/35) | | 10.3 (4/39) | 2.6 (1/39) | 37.1 (13/35) | | 28.2 (11/39) | 75.0 (3/4) | 26.9 (7/26) | 54.8 (23/42) | 38.5 (10/26) | 11.9 (5/42) | 34.6 (9/26) | 33.3 (14/42) | NO |  |
| Assessment of liquor for meconium | 87.5 (35/40) | 79.1 (34//43) | 100.0 (8/8) | 0.0 (0 (5/40) | | 11.6 (5/43) | 0.0 (0/8) | 12.5 (5/40) | | 9.3 (4/43) | 0.0 (0/8) |  | | | | | | | | | | | | | | | | | IN |  |
| Assessment of foetal movements by maternal perception | 70.3 (26/37) | 25.0 (10/40) | 62.5 (5/8) | 18.9 (7/37) | | 20.0 (8/40) | 12.5 (1/8) | 10.8 (4/37) | | 55.0 (22/40) | 25.0 (2/8) | 85.7 (30/35) | 30.8 (12/39) | 50.0 (2/4) | 8.6 (3/35) | | 23 (9/39) | 25.0 (1/4) | 5.7 (2/35) | | 46.2 (18/39) | 25.0 (1/4) | 80.8 (21/26) | 38.1 (16/42) | 7.7 (2/26) | 11.9 (5/42) | 11.5 (3/26) | 50.0 (21/42) | NO |  |
| Assessment of foetal movement by ultrasound detection | 45.7 (16/35) | 9.8 (4/41) | 62.5 (5/8) | 20.0 (7/35) | | 9.8 (4/41) | 25.0 (2/8) | 34.3 (12/35) | | 80.5 (33/41) | 12.5 (1/8) | 34.2 (12/35) | 7.7 (3/39) | 75.0 (3/4) | 11.4 (4/35) | | 12.8 (5/39) | 25.0 (1/4) | 54.3 (19/35) | | 79.5 (31/39) | 0.0 (0/4) | 19.2 (5/26) | 0.0 (0/42) | 26.9 (7/26) | 11.9 (5/42) | 53.8 (14/26) | 88.1 (37/42) | NO |  |
| Foetal Acoustic Stimulation Test | 30.3 (10/33) | 27.5 (11/40) | 12.5 (1/8) | 30.3 (10/33) | | 10.0 (4/40) | 25.0 (2/8) | 39.4 (13/33) | | 62.5 (25/40) | 62.5 (5/8) | 17.1 (6/35) | 17.9 (7/39) | 0.0 (0/4) | 28.6 (10/35) | | 12.8 (5/39) | 25.0 (1/4) | 54.3 (19/35) | | 69.2 (27/39) | 75.0 (3/4) | 7.7 (2/26) | 14.3 (6/42) | 23.1 (6/26) | 4.8 (2/42) | 69.2 (18/26) | 81.0 (34/42) | OUT** |  |
| None: No additional test, continue monitoring | 37.9 (11/29) | 25.0 (10/40) | 25.0 (2/8) | 3.4 (1/29) | | 12.5 (5/40) | 12.5 (1/8) | 58.6 (17/29) | | 62.5 (25/40) | 62.5 (5/8) | 25.7 (9/35) | 23.1 (9/39) | 0.0 (0/4) | 11.4 (4/35) | | 5.1  (2/39) | 25.0 (1/4) | 62.9 (22/35) | | 71.8 (28/39) | 75.0 (3/4) | 15.4 (4/26) | 26.2 (11/42) | 11.5 (3/26) | 11.9 (5/42) | 73.1 (19/26) | 61.9 (25/42) | NO |  |
| None: No additional test, immediate delivery | 14.3 (4/28) | 20.0 (7/35) | 12.5 (1/8) | 17.9 (5/28) | | 14.3 (5/35) | 37.5 (3/8) | 67.9 (19/28) | | 65.7 (23/35) | 50.0 (4/8) | 31.4 (11/35) | 10.3 (4/39) | 25.0 (1/4) | 11.4 (4/35) | | 2.6 (1/39) | 0.0 (0/4) | 57.1 (20/35) | | 87.2 (34/39) | 75.0 (3/4) | 11.5 (3/26) | 4.8 (2/42) | 11.5 (3/26) | 9.5 (4/42) | 76.9 (20/26) | 85.7 (36/42) | OUT |  |
| Intrauterine resuscitation* |  | | | | | | | | | | | 82.3 (29/35) | 94.9 (37/39) | 75.0 (3/4) | 14.3 (5/35) | | 0.0 (0/39) | 0.0 (0/4) | 2.9 (1/35) | | 5.1 (2/39) | 25.0 (1/4) |  | | | | | | IN |  |
| Biophysical Profile* |  |  |  |  |  |  |  |  |  |  |  | 14.3 (5/35) | 23.1 (9/39) | 25.0 (1/4) | 14.3 (5/35) | | 5.1 (2/39 ( | 25.0 (1/4) | 31.4 (11/35) | | 76.9 (30/39) | 75.0 (3/4) | 26.9 (7/26) | 2.4 (1/42) | 19.2 (5/26) | 9.5 (4/42) | 53.8 (14/26) | 88.1 (37/42) | NO |  |
| In case foetal heart beat is suboptimal, what adjunctive test (s) would you do to confirm foetal well-being in the second stage of active phase of labour? | | | | | | | | | | | | | | | | | | | | | | | | | | | | |  |  |
| Foetal Scalp Sampling | 40.6 (13/32) | 30.0 (12/40) | 37.5 (3/8) | 12.5 (4/32) | | 10.0 (4/40) | 12.5 (1/8) | 46.9 (15/32) | | 60.0 (24/40) | 50.0 (4/8) | 14.7 (5/34) | 23.1 (9/39) | 25.0 (1/4) | 14.7 (5/34) | | 5.1 (2/39) | 0.0 (0/4) | 70.5 (24/34) | | 71.8 (28/39) | 75.0 (3/4) | 3.8  (1/26) | 16.7 (7/42) | 19.2 (5/26) | 2.4 (1/42) | 76.9 (20/26) | 81.0 (34/42) | OUT** |  |
| Foetal Pulse Oximetry | 16.7 (5/30) | 12.8 (5/39) | 25.0 (2/8) | 30.0 (9/30) | | 10.3 (4/39) | 0.0 (0/8) | 53.3 (16/30) | | 76.9 (30/39) | 75.0 (6/8) | 11.8 (4/34) | 5.1 (2/39) | 25.0 (1/4) | 20.6 (7/34) | | 5.1 (2/39) | 0.0 (0/4) | 67.6 (23/34) | | 89.7  (35/39) | 75.0 (3/4) | 15.4 (4/26) | 2.4 (1/42) | 11.5 (3/26) | 4.8 (2/42) | 73.1 (19/26) | 92.9 (39/42) | OUT** |  |
| Foetal Scalp Stimulation Test | 17.2 (5/29) | 39.0 (16/41) | 0.0 (0/8) | 24.1 (7/29) | | 17.1 (7/41) | 12.5 (1/8) | 58.6 (17/29) | | 43.9 (18/41) | 87.5 (7/8) | 20.5 (7/34) | 38.5 (15/39) | 0.0 (0/4) | 17.6 (6/34) | | 10.3  (4/39) | 0.0 (0/4) | 61.8 (21/34) | | 51.3 (20/39) | 100.0 (4/4) | 7.7  (2/26) | 23.8 (10/42) | 23.1 (6/26) | 14.3 (6/42) | 69.2 (18/26) | 61.9 (26/42) | NO |  |
| Assessment of liquor for meconium | 85.7 (30/35) | 61.0 (25/41) | 7.5 (7/8) | 2.9 (1/35) | | 12.2 (5/41) | 0.0 (0/8) | 11.4 (4/35 | | 26.8 (11/41) | 12.5 (1/8) | 88.2 (30/34) | 66.7 (26/39) | 75.0 (3/4) | 8.8 (3/34) | | 7.7 (3/39) | 0.0 (0/4) | 2.9 (1/34) | | 25.6 (10/39) | 25.0 (1/4) | 96.2 (25/26) | 85.7 (25/42) | 3.8 (1/26) | 7.1 (3/42) | 0.0 (0/26) | 7.1 (3/42) | IN |  |
| Assessment of foetal movements by maternal perception | 68.6 (24/35) | 10.3 (4/39) | 57.1 (4/7) | 11.4 (4/35) | | 28.2 (11/39) | 0.0 (0/7) | 20.0 (7/35) | | 61.5 (24/39) | 42.9 (3/7) | 58.8 (20/34) | 10.3 (4/39) | 50.0 (2/4) | 20.6 (7/34) | | 15.4 (6/39) | 25.0 (1/4) | 20.6 (7/34) | | 74.4 (29/39) | 25.0 (1/4) | 73.1 (19/26) | 9.8 (4/42) | 7.7 (2/26) | 12.2 (5/42) | 19.2 (5/26) | 78.0 (32//42) | NO |  |
| Assessment of foetal movement by ultrasound detection | 32.3 (10/31) | 2.5 (1/40) | 37.5 (3/8) | 19.4 (6/31) | | 12.5 (5/40) | 12.5 (1/8) | 48.4 (15/31) | | 85.0 (34/40) | 50.0 (4/8) | 26.5 (9/34) | 0 .0  (0/39) | 25.0 (1/4) | 8.8 (3/34) | | 2.6 (1/39) | 25.0 (1/4) | 64.7 (22/34) | | 97.4 (38/39) | 50.0 (2/4) | 19.2 (5/26) | 0.0 (0/42) | 15.4 (4/26) | 4.8 (2/42) | 65.4 (17/26) | 95.2 (40/42) | NO |  |
| Foetal Acoustic Stimulation Test | 23.3 (7/30) | 10.3 (4/39) | 0.0 (0/8) | 30.0 (9/30) | | 7.7 (3/39) | 25.0 (2/8) | 46.7 (14/30) | | 82.1 (32/39) | 75.0 (6/8) | 17.6 (6/34) | 12.8 (5/39) | 0.0 (0/4) | 11.8 (4/34) | | 7.7 (3/39) | 25.0 (1/4) | 70.6 (24/34) | | 79.5  (31/39) | 75.0 (3/4) | 3.8 (1/26) | 9.5 (4/42) | 26.9 (7/26) | 4.8 (2/42) | 69.2 (18/26) | 85.7 (36/42) | OUT** |  |
| None: No additional test, continue monitoring | 32.1 (9/28) | 24.3 (9/37) | 25.0 (2/8) | 17.9 (5/28) | | 10.8 (4/37) | 12.5 (1/8) | 50.0 (14/28) | | 64.9 (24/37) | 62.5 (5/8) | 29.4 (10/34) | 17.9 (7/39) | 0.0  (0/4) | 11.8 (4/34) | | 10.3 (4/39) | 25.0 (1/4) | 58.8 (20/34) | | 71.8 (28/39) | 75.0 (3/4) | 15.4 (4/26) | 21.4 (9/42) | 15.4 (4/26) | 11.9 (5/42) | 69.2 (18/26) | 66.7 (28/42) | NO |  |
| None: No additional test, immediate delivery | 50.0 (17/17) | 43.9 (18/41) | 25.0 (2/8) | 11.8 (4/17) | | 14.6 (6/41) | 12.5 (1/8) | 38.2 (13/17) | | 41.5 (17/41) | 62.5 (5/8) | 50.0 (17/34) | 53.8 (21/39) | 50.0 (2/4) | 18.6 (6/34) | | 0.0 (0/39) | 0.0 (0/4) | 32.4 (11/34) | | 46.2 (18/39) | 50.0 (2/4) | 15.4 (4/26) | 21.4 (9/42) | 23.1 (6/26) | 16.7 (7/42) | 61.5 (16/26) | 61.9 (26/42) | NO |  |
| Intrauterine resuscitation* |  | | | | | | | | | | | 85.3 (29/34) | 74.4 (29/39) | 75.0 (3/4) | 2.9 (1/34) | | 10.3 (4/39) | 25.0 (1/4) | 11.8 (4/34) | | 15.4 (6/39) | 0.0  (0/4) | 88.5 (23/26) | 88.1 (37/42) | 3.8 (1/26) | 7.1 (3/42) | 7.7 (2/26) | 4.8 (2/42) | IN |  |
| Biophysical Profile* |  |  |  |  |  |  |  |  |  |  |  | 2.9 (1/34) | 0.0 (0/39) | 25.0 (1/4) | 17.6 (6/34) | | 5.1 (2/39) | 50.0 (2/4) | 50.0 (17/34) | | 94.9121212 (37/39) | 25.0 (1/4) | 15.4 (4/26) | 0.0 (0/42) | 23.1 (6/26) | 2.4 (1/42) | 61.5 (16/26) | 97.6 (41/42) | NO |  |
| In case foetal heart beat is abnormal, what adjunctive test (s) would you do to confirm foetal well-being in the first stage of active phase of labour? | | | | | | | | | | | | | | | | | | | | | | | | | | | | |  |  |
| Foetal Scalp Sampling | 51.6 (16/31) | 42.5 (17/40) | 25.0 (2/8) | 9.7 (3/31) | | 10.0 (4/40) | 12.5 (1/8) | 38.7 (12/31) | | 47.5 (19/40) | 62.5 (5/8) | 36.4 (12/33) | 53.8 (21/39) | 25.0 (1/4) | 9.1 (3/33) | | 7.7 (3/39) | 0.0 (0/4) | 54.5 (18/33) | | 38.5 (15/39) | 75.0 (3/4) | 50.0 (13/26) | 45.2 (19/42) | 11.5 (2/26) | 7.1 (3/42) | 38.5 (10/26) | 47.6 (20/42) | NO |  |
| Foetal Pulse Oximetry | 31.0 (9/29) | 15.8 (6/38) | 37.5 (3/8) | 24.1 (7/29) | | 10.5 (4/38) | 12.5 (1/8) | 44.8 (13/29) | | 73.7 (28/38) | 50.0 (4/8) | 21.2 (7/33) | 7.7 (3/39) | 0.0 (0/4) | 21.2 (7/33) | | 12.8 (5/39) | 0.0 (0/4) | 57.6 (19/33) | | 79.5 (31/39) | 100.0 (4/4) | 23.1 (6/26) | 0.0 (0/42) | 11.5 (3/26) | 11.9 (5/42) | 65.4 (17/26) | 88.1 (17/42) | NO |  |
| Foetal Scalp Stimulation Test | 21.4 (6/28) | 47.5 (19/40) | 25.0 (2/8) | 32.1 (9/28) | | 10.0 (4/40) | 12.5 (1/8) | 46.4 (13/28) | | 42.5 (17/40) | 62.5 (5/8) | 12.1 (4/33) | 56.4 (22/39) | 0.0 (0/4) | 21.2 (7/33) | | 10.3 (4/39) | 0.0 (0/4) | 66.7 (22/33) | | 33.3 (13/39) | 100.0 (4/4) | 15.4 (4/26) | 45.2 (19/42) | 26.9 (7/26) | 11.9 (5/42) | 57.7 (15/26) | 42.9 (18/42) | NO |  |
| Assessment of liquor for meconium | 78.8 (26/33) | 60.0 (24/40) | 62.5 (5/8) | 6.1 (2/33) | | 15.0 (6/40) | 25.0 (2/8) | 15.2 (5/33) | | 25.0 (10/40) | 12.5 (1/8) | 87.9 (29/33) | 71.8 (28/39) | 75.0 (3/4) | 9.1 (3/33) | | 15.4 (6/39) | 0.0 (0/4) | 3.0 (1/33) | | 12.8 (5/39) | 25.0 (1/4) |  | | | | | | IN |  |
| Assessment of foetal movements by maternal perception | 62.5 (20/32) | 17.9 (7/39) | 50.0 (4/8) | 25.0 (8/32) | | 17.9 (7/39) | 25.0 (2/8) | 12.5 (4/32) | | 64.1 (25/39) | 25.0 (2/8) | 69.7  (23/33) | 12.8 (5/39) | 50.0 (2/4) | 12.1 (4/33) | | 15.4 (6/39) | 25.0 (1/4) | 18.2 (6/33) | | 71.8 (28/39) | 25.0 (1/4) | 84.6 (22/26) | 12.2 (5/42) | 3.8 (1/26) | 4.9 (2/42) | 11.5 (3/26) | 82.9 (34/42) | NO |  |
| Assessment of foetal movement by ultrasound detection | 61.8 (21/34) | 5.3 (2/38) | 37.5 (3/8) | 5.9 (2/34) | | 10.5 (4/38) | 25.0 (2/8) | 32.4 (11/34) | | 84.2 (32/38) | 37.5 (3/8) | 36.4 (12/33) | 0.0 (0/39) | 50.0 (2/4) | 21.2 (7/33) | | 7.7 (3/39) | 25.0 (1/4) | 42.2 (14/33) | | 92.3 (36/39) | 25.0 (1/4) | 26.9 (7/26) | 0.0 (0/42) | 15.4 (4/26) | 2.4 (4/42) | 57.7 (15/26) | 97.6 (40/42) | NO |  |
| Foetal Acoustic Stimulation Test | 33.3 (10/30) | 15.8 (6/38) | 0.0 (0/8) | 33.3 (10/30) | | 10.5 (4/38) | 25.0 (2/8) | 33.3 (10/30) | | 73.7 (28/38) | 75.0 (6/8) | 6.1 (2/33) | 20.5 (8/39) | 0 (0/4) | 30 (10/33) | | 8 (3/39) | 0 (0/4) | 64 (21/33) | | 72 (28/39) | 100.0 (4/4) | 11.5 (3/26) | 7.1 (3/42) | 19.2 (5/26) | 2.4 (1/42) | 69.2 (18/26) | 90.5 (38/42) | OUT** |  |
| None: No additional test, continue monitoring | 33.3 (9/27) | 14.3 (5/35) | 0.0 (0/8) | 11.1 (3/27) | | 8.6 (3/35) | 12.5 (1/8) | 55.6 (15/27) | | 77.1 (27/35) | 87.5 (7/8) | 9.1 (3/33) | 15.4  (6/39) | 0 (0/4) | 12 (4/33) | | 8 (3/39) | 25 (1/4) | 79 (26/33) | | 77 (30/39) | 75.0 (3/4) | 12.0 (3/26) | 9.5 (4/42) | 8.0 (2/26) | 2.4 (1/42) | 80.0 (20/26) | 88.1 (37/42) | OUT |  |
| None: No additional test, immediate delivery | 46.9 (15/32) | 56.1 (23/41) | 37.5 (3/8) | 12.5 (4/32) | | 14.6 (6/41) | 12.5 (1/8) | 40.6 (13/32) | | 29.3 (12/41) | 50.0 (4/8) | 39.4 (13/33) | 59.0 (23/39) | 75 (3/4) | 19 (6/33) | | 5 (2/39) | 25 (1/4) | 41 (13/33) | | 36 (14/39) | 0.0  (0/4) | 46.2 (12/26) | 73.8 (31/42) | 15.4 (4/26) | 7.1 (3/42) | 38.5 (10/26) | 19.0 (8/42) | NO |  |
| Intrauterine resuscitation* |  | | | | | | | | | | | 81.2 (27/33) | 92.3 (36/39) | 75 (3/4) | 12 (4/33) | | 3 (1/39) | 25 (1/4) | 6 (2/33) | | 5 (2/39) | 0.0  (0/4) |  | | | | | | IN |  |
| Biophysical Profile* |  |  |  |  |  |  |  |  |  |  |  | 39.4 (13/33) | 10.3 (4/39) | 0 (0/4) | 9 (3/32) | | 5 (2/39) | 50 (2/4) | 50 (16/33) | | 84 (32/39) | 50.0 (2/4) | 19.2 (5/26) | 2.4 (1/42) | 15.4 (4/26) | 2.4 (1/42) | 65.4 (17/26) | 95.2 (40/42) | NO |  |
| In case foetal heart beat is abnormal, what adjunctive test (s) would you do to confirm foetal well-being in the second stage of active phase of labour? | | | | | | | | | | | | | | | | | | | | | | | | | | | | |  |  |
| Foetal Scalp Sampling | 45.2 (14/31) | 36.8 (14/38) | 28.6 (2/7) | 12.9 (4/31) | | 12.9 (4/31) | 5.3 (2/38) | 41.9 (13/31) | | 57.9 (22/38) | 57.9 (22/38) | 15.2 (5/33) | 25.6 (10/39) | 25.0 (1/4) | 12.1 (4/33) | | 10.3 (4/39) | 0.0 (0/4) | 72.7 (24/33) | | 64.1 (25/39) | 75.0 (3/4) | 15.4 (4/26) | 21.4 (9/42) | 11.5 (3/26) | 2.4 (1/42) | 73.1 (19/26) | 76.2 (32/42) | NO |  |
| Foetal Pulse Oximetry | 41.4 (12/29) | 18.9 (7/37) | 0.0 (0/7) | 17.2 (5/29) | | 5.4 (2/37) | 28.6 (2/7) | 41.4 (12/29) | | 75.7 (28/37) | 71.4 (5/7) | 18.1 (6/33) | 5.1 (2/39) | 0.0 (0/4) | 18.2 (6/33) | | 7.7 (3/39) | 25.0 (1/4) | 63.4 (21/33) | | 87.2 (34/39) | 75.0 (3/4) | 11.5 (3/26) | 2.4 (1/42) | 11.5 (3/26) | 0.0 (0/42) | 76.9 (20/26) | 97.6 (41/42) | OUT |  |
| Foetal Scalp Stimulation Test | 34.5 (10/29) | 39.5 (15/38) | 14.3 (1/7) | 20.7 (6/29) | | 10.5 (4/38) | 14.3 (1/7) | 44.8 (13/29) | | 50.0 (19/38) | 71.4 (5/7) | 12.1 (4/33) | 30.8 (12/39) | 0.0 (0/4) | 15.2 (5/33) | | 2.6 (1/39) | 25.0 (1/4) | 72.7 (24/33) | | 66.7 (26/39) | 75.0 (3/4) | 3.8  (1/26) | 21.4 (9/42) | 26.9 (7/26) | 4.8 (2/42) | 69.2 (18/26) | 73.8 (31/42) | NO |  |
| Assessment of liquor for meconium | 78.1 (25/32) | 56.4 (22/39) | 71.4 (5/7) | 6.3 (2/32) | | 7.7 (3/39) | 14.3 (1/7) | 15.6 (5/32) | | 35.9 (14/39) | 14.3 (1/7) | 90.1 (30/33) | 66.7 (26/39) | 75.0 (3/4) | 3.0 (1/33) | | 10.3 (4/39) | 25.0 (1/4) | 6.1 (2/33) | | 23.1 (9/39) | 0.0 (0/4) | 96.2 (25/26) | 66.7 (28/42) | 3.8 (1/26) | 7.1 (3/42) | 0.0 (0/26) | 26.2 (11/42) | NO |  |
| Assessment of foetal movements by maternal perception | 54.8 (17/31) | 8.1 (3/37) | 42.9 (3/7) | 19.4 (6/31) | | 24.3 (9/37) | 14.3 (1/7) | 25.8 (8/31) | | 67.6 (25/37) | 42.9 (3/7) | 60.1 (20/33) | 12.8 (5/39) | 25.0 (1/4) | 12.1 (4/33) | | 7.7 (3/39) | 50.0 (2/4) | 27.3 (9/33) | | 79.5 (31/39) | 25.0 (1/4) | 73.1 (19/26) | 4.8 (2/42) | 3.8 (1/26) | 2.4 (1/42) | 23.1 (6/26) | 92.9 (39/42) | NO |  |
| Assessment of foetal movement by ultrasound detection | 44.8 (13/29) | 2.7 (1/37) | 28.6 (2/7) | 13.8 (4/29) | | 16.2 (6/37) | 42.9 (3/7) | 41.4 (12/29) | | 81.1 (30/37) | 28.6 (2/7) | 30.3 (10/33) | 0.0 (0/39) | 25.0 (1/4) | 12.1  (4/33) | | 5.1 (2/39) | 50.0 (2/4) | 57.6 (19/33) | | 94.8 (37/39) | 25.0 (1/4) | 15.4 (4/26) | 0.0 (0/42) | 11.5 (3/26) | 4.8 (2/42) | 73.1 (19/26) | 95.2 (40/42) | OUT** |  |
| Foetal Acoustic Stimulation Test | 27.6 (8/29) | 13.5 (5/37) | 0.0 (0/7) | 24.1 (7/29) | | 8.1 (3/37) | 42.9 (3/7) | 48.3 (14/29) | | 78.4 (29/37) | 57.1 (4/7) | 12.1 (4/33) | 12.8 (5/39) | 0.0 (0/4) | 15.2 (5/33) | | 2.6 (1/39) | 50.0 (2/4) | 72.7 (24/33) | | 84.6 (33/39) | 50.0 (2/4) |  | | | | | | OUT |  |
| None: No additional test, continue monitoring | 22.2 (6/27) | 8.6 (3/35) | 0.0 (0/7) | 11.1 (3/27) | | 11.4 (4/35) | 14.3 (1/7) | 66.7 (18/27) | | 80.0 (28/35) | 85.7 (6/7) | 24.2 (8/33) | 12.8 (5/39) | 0.0 (0/4) | 18.2 (6/33) | | 7.7 (3/39) | 25.0 (1/4) | 57.6 (19/33) | | 79.5 (31/39) | 75.0 (3/4) | 11.5 (3/26) | 2.4 (1/42) | 11.5 (3/26) | 7.1 (3/42) | 76.9 (20/26) | 90.5 (20/42) | OUT |  |
| None: No additional test, immediate delivery | 57.1 (20/35) | 71.1 (32/45) | 50.0 (4/8) | 2.9 (1/35) | | 13.3 (6/45) | 12.5 (1/8) | 40.0 (14/35) | | 15.6 (7/45) | 37.5 (3/8) | 78.8 (26/33) | 82.1 (32/39) | 100.0 (4/4) | 12.1 (4/33) | | 0.0 (0/39) | 0.0 (0/4) | 9.1 (3/33) | | 17.9 (7/39) | 0.0  (0/4) | 80.8 (21/26) | 83.3 (35/42) | 7.7 (2/26) | 2.4 (1/42) | 11.5 (3/26) | 14.3 (5/42) | IN |  |
| Intrauterine resuscitation* |  | | | | | | | | | | | 84.8 (28/33) | 76.9 (30/39) | 75.0 (3/4) | 6.1 (2/33) | | 7.7 (3/39) | 25.0 (0/4) | 9.1 (3/33) | | 15.4 (6/39) | 0.0  (0/4) | 96.2 (25/26) | 88.1 (37/42) | 3.8 (1/26) | 4.8 (2/42) | 0.0 (0/26) | 7.1 (3/42) | IN |  |
| Biophysical Profile* |  |  |  |  |  |  |  |  |  |  |  | 33.3 (11/33) | 0.0 (0/39) | 0.0 (0/4) | 9.1 (3/33) | | 5.1 (2/39) | 50.0 (2/4) | 57.6 (19/33) | | 94.9 (37/39) | 50.0  (2/4) | 11.5 (3/26) | 0.0 (0/42) | 19.2 (5/26) | 4.8 (2/42) | 69.2 (18/26) | 95.2 (40/42) | OUT** |  |
| **Legend:**  Numbers are in % (n)  M= Midwives, O=Obstetricians, P = Paediatricians; The paediatric was deemed too small and hence consensus in round 2 and 3 was based on midwife and obstetrician groups.  *New option, ** Outcomes that nearly reached consensus and were discussed by the steering committee for a final decision | | | | | | | | | | | | | | | | | | | | | | | | | | | | | |  |
