# Supplementary material for: Delphi consensus statement on intrapartum fetal monitoring in low‐resource settings
Source: Int J Gynaecol Obstet. 2018 Dec 24;146(1):8–16. doi: 10.1002/ijgo.12724 (PMC7379246; doi:10.1002/ijgo.12724)
Supplement: Supplementary file 2 — Table S2. Results per stakeholder group and per round (multiple choice questions). [file IJGO-146-8-s002.docx]

| Table S2: Results per stakeholder group and per round (Multiple-choice questions) | | | | | | | | | | | | | | |
| --- | --- | --- | --- | --- | --- | --- | --- | --- | --- | --- | --- | --- | --- | --- |
| Question | **Round** | **Outcomes per stakeholder group (%)** | | | | | | | | | | | | **CONSENSUS IN (YES/NO)** |
| By Intermittent Auscultation, what would be the minimal acceptable frequency of foetal heart rate monitoring for low maternal and low foetal risk pregnancies during the first stage of active phase of labour? |  | Stakeholder group* | Every 15 minutes | Every 30 minutes | Every 60 minutes | Every 90 minutes | Every 2 hours | Other | Before 6 cm:  every 4 hours; after 6 cm: every one hour* | | | | YES (EVERY 30 MINUTES) | |
|  | ROUND 1 | M (n=45) | 13.3 | 68.9 | 11.1 | 0.0 | 4.4 | 2.2 | | |  | |  |  |
|  |  | O (n=47) | 17.0 | 44.7 | 19.1 | 2.1 | 10.6 | 6.4 | | |  |  |  |  |
|  |  | P (n=8) | 0.0 | 25.0 | 37.5 | 0.0 | 37.5 | 6.4 | | |  |  |  |  |
|  | ROUND 2 | M (n=36) | 5.6 | 63.9 | 11.1 |  | 0.0 | 11.1 | | | 8.3 | |  |  |
|  |  | O (n=41) | 4.9 | 58.5 | 14.6 |  | 0.0 | 7.3 | | | 14.6 | |  |  |
|  |  | P (n=8) | 0.0 | 25.0 | 50.0 |  | 0.0 | 0.0 | | | 25.0 | |  |  |
|  | ROUND 3 | M (n=26) |  | 84.6 | 3.8 |  |  |  | | | 11.5 | |  |  |
|  |  | O (n=42) |  | 71.4 | 19.0 |  |  |  |  |  | 9.5 | |  |  |
| By Intermittent Auscultation, what would be the minimal acceptable frequency of foetal heart rate monitoring of low maternal and low foetal risk pregnancies during the second stage of active phase of labour? |  |  | After every contraction | Every 5 minutes | Every 15 minutes | Every 30 minutes | others |  | | | | | NO | |
|  | ROUND 1 | M (n=45) | 40.0 | 26.7 | 26.7 | 4.4 | 2.2 |  | | | | |  |  |
|  |  | O (n=47) | 27.7 | 29.8 | 29.8 | 10.6 | 2.1 |  |  |  |  |  |  |  |
|  |  | P (n=8) | 0.0 | 12.5 | 50.0 | 37.5 | 0.0 |  |  |  |  |  |  |  |
|  | ROUND 2 | M (n=36) | 50.0 | 5.6 | 27.8 | 8.3 | 8.3 |  |  |  |  |  |  |  |
|  |  | O (n=41) | 36.6 | 39.0 | 19.5 | 0.0 | 4.9 |  |  |  |  |  |  |  |
|  |  | P (n=4) | 0.0 | 0.0 | 100.0 | 0.0 | 0.0 |  |  |  |  |  |  |  |
|  | ROUND 3 | M (n=26) | 73.1 | 19.2 | 7.7 |  |  |  |  |  |  |  |  |  |
|  |  | O (n=42) | 50.0 | 35.7 | 14.3 |  |  |  |  |  |  |  |  |  |
| By Intermittent Auscultation, how long would you listen to the foetal heart rate for low maternal risk and low foetal risk pregnancies during labour? |  |  | 10 seconds | 15 seconds | 30 seconds | 60 seconds | Others |  |  |  |  |  | NO | |
|  | ROUND 1 | M (n=45) | 6.7 | 15.6 | 15.6 | 55.6 | 6.7 |  |  |  |  |  |  |  |
|  |  | O (n=47) | 4.3 | 14.9 | 42.6 | 31.9 | 6.4 |  |  |  |  |  |  |  |
|  |  | P (n=8) | 12.5 | 0.0 | 12.5 | 62.5 | 12.55 |  |  |  |  |  |  |  |
|  | ROUND 2 | M (n=36) |  | 0.0 | 38.9 | 50.0 | 11.1 |  |  |  |  |  |  |  |
|  |  | O (n=41) |  | 14.6 | 43.9 | 39.0 | 2.4 |  |  |  |  |  |  |  |
|  |  | P (n=4) |  | 0.0 | 25.0 | 75.0 | 0.0 |  |  |  |  |  |  |  |
|  | ROUND 3 | M (n=26) |  | 3.8 | 42.3 | 53.8 |  |  |  |  |  |  |  |  |
|  |  | O (n=42) |  | 9.5 | 52.4 | 38.1 |  |  |  |  |  |  |  |  |
| What would be the minimal acceptable frequency of contraction monitoring in low maternal and low foetal risk pregnancies in the first stage of active phase of labour? |  |  | Every 30 minutes | Every 1 hour | Every 90 minutes | Every 2 hours | Every 3 hours | Every 4 hours | | Other | | No monitoring of contractions in first stage. If inadequate dilatation after 4 hours, assess if this is due to poor contractions* | YES (EVERY 1 HOUR) | |
|  | ROUND 1 | M (n=45) | 51.1 | 28.9 | 4.4 | 6.7 | 0.0 | 2.2 | | 6.7 | |  |  |  |
|  |  | O (n=47) | 19.1 | 48.9 | 4.3 | 19.1 | 2.1 | 2.1 | | 4.3 | |  |  |  |
|  |  | P (n=8) | 37.5 | 25.0 | 0.0 | 37.5 | 0.0 | 0.0 | | 0.0 | |  |  |  |
|  | ROUND 2 | M (n=36) | 33.3 | 41.7 |  | 8.3 |  |  | | 2.8 | | 13.9 |  |  |
|  |  | O (n=41) | 12.2 | 61.0 |  | 12.2 |  |  | | 2.4 | | 12.2 |  |  |
|  |  | P (n=4) | 0.0 | 50.0 |  | 25.0 |  |  | | 0.0 | | 25.0 |  |  |
|  | ROUND 3 | M (n=26) | 19.2 | 80.8 |  | 0.0 |  |  | |  | | 0.0 |  |  |
|  |  | O (n=42) | 11.9 | 88.1 |  | 0.0 |  |  | |  | | 0.0 |  |  |
| For how many minutes would you feel for contractions in low maternal and low foetal risk pregnancies during active phase of labour? |  |  | 5 minutes | 10 minutes | Other | 5 minutes when not on medication stimulation and 10 minutes if on induction/augmentation protocol.* |  |  | | | | | YES (10 MINUTES) | |
|  | ROUND 1 | M (n=45) | 20.0 | 71.1 | 8.9 |  |  |  | | | | |  |  |
|  |  | O (n=47) | 29.8 | 66.0 | 4.3 |  |  |  |  |  |  |  |  |  |
|  |  | P (n=8) | 62.5 | 12.5 | 25.0 |  |  |  |  |  |  |  |  |  |
|  | ROUND 2 | M (n=36) | 8.3 | 66.7 | 0.0 | 25.0 |  |  |  |  |  |  |  |  |
|  |  | O (n=41) | 19.5 | 61.0 | 0.0 | 19.5 |  |  |  |  |  |  |  |  |
|  |  | P (n=4) | 75.0 | 0.0 | 0.0 | 25.0 |  |  |  |  |  |  |  |  |
|  | ROUND 3 | M (n=26) | 11.5 | 88.5 |  | 0.0 |  |  |  |  |  |  |  |  |
|  |  | O (n=42) | 26.2 | 73.8 |  | 0.0 |  |  |  |  |  |  |  |  |
| By Intermittent Auscultation, what would be the minimal acceptable frequency of FHR in low maternal, high foetal risk pregnancies during the first stage of active phase of labour? |  |  | Every 15 minutes | Every 30 minutes | Every 1 hour | Every 2 hours | Other |  | | | | | NO | |
|  | ROUND 1 | M (n=43) | 51.2 | 44.2 | 4.7 | 0.0 | 0.0 |  | | | | |  |  |
|  |  | O (n=47) | 59.6 | 29.8 | 8.5 |  | 2.1 |  |  |  |  |  |  |  |
|  |  | P (n=8) | 50.0 | 25 | 12.5 | 12.5 | 0.0 |  |  |  |  |  |  |  |
|  | ROUND 2 | M (n=35) | 60.0 | 40.0 |  | | 0.0 |  |  |  |  |  |  | |
|  |  | O (n=39) | 59.0 | 33.3 |  |  | 7.7 |  |  |  |  |  |  |  |
|  |  | P (n=4) | 25.0 | 75.0 |  |  | 0.0 |  |  |  |  |  |  |  |
|  | ROUND 3 | M (n=26) | 65.4 | 34.6 |  |  |  |  |  |  |  |  |  |  |
|  |  | O (n=42) | 73.8 | 26.2 |  |  |  |  |  |  |  |  |  |  |
| By Intermittent Auscultation, what would be the minimal acceptable frequency of FHR in low maternal, high foetal risk pregnancies during the second stage of active phase of labour? |  |  | After every contraction | After every 5 minutes | After every 15 minutes | After every 30 minutes | Other |  | | | | | YES  (AFTER EVERY CONTRACTION) | |
|  | ROUND 1 | M (n=43) | 62.8 | 20.9 | 14.0 | 2.3 | 0.0 |  | | | | |  |  |
|  |  | O (n=47) | 63.8 | 17.0 | 10.6 | 4.3 | 4.3 |  |  |  |  |  |  |  |
|  |  | P (n=8) | 25.0 | 12.5 | 50.0 | 0.0 | 12.5 |  |  |  |  |  |  |  |
|  | ROUND 2 | M (n=35) | 77.1 | 11.4 | 8.6 |  | 2.9 |  |  |  |  |  |  |  |
|  |  | O (n=39) | 74.4 | 12.8 | 7.7 |  | 5.1 |  |  |  |  |  |  |  |
|  |  | P (n=4) | 25.0 | 25.0 | 50.0 |  | 0 |  |  |  |  |  |  |  |
| By Intermittent Auscultation, how long would you listen to the foetal heart rate for in low maternal and high risk pregnancies during active labour? |  |  | 60 seconds | 30 seconds | 15seconds | 10 seconds | Others |  | | | | | YES (60 SECONDS) | |
|  | ROUND 1 | M (n=43) | 72.1 | 11.6 | 7.0 | 4.7 | 4.7 |  | | | | |  |  |
|  |  | O (n=47) | 57.4 | 34.7 |  |  | 6.4 |  |  |  |  |  |  |  |
|  |  | P (n=8) | 50.0 | 50.0 |  |  |  |  |  |  |  |  |  |  |
|  | ROUND 2 | M (n=35) | 82.9 | 11.4 |  |  | 5.7 |  |  |  |  |  |  |  |
|  |  | O (n=39) | 74.4 | 20.5 |  | 5.1 |  |  |  |  |  |  |  |  |
|  |  | P (n=4) | 100.0 | 0.0 |  | 0.0 |  |  |  |  |  |  |  |  |
| What would be the minimal acceptable frequency of contraction monitoring in low maternal and high foetal risk pregnancies in the first stage of active phase of labour? |  |  | Every 30 minutes | Every 1 hour | Every 90 minutes | Every 2 hours | Every 4 hours | other | | | No monitoring of contractions in first stage. If inadequate dilatation after 4 hours, assess if this is due to poor contraction)* | | NO | |
|  | ROUND 1 | M (n=43) | 74.4 | 20.9 | 0.0 | 0.0 | 0.0 | 4.7 | | |  | |  |  |
|  |  | O (n=47) | 36.2 | 42.6 | 4.3 | 12.8 | 0.0 | 4.3 | | |  |  |  |  |
|  |  | P (n=8) | 37.5 | 37.5 | 0.0 | 12.5 | 0.0 | 12.5 | | |  |  |  |  |
|  | ROUND 2 | M (n=35) | 60.0 | 25.7 |  |  |  | 2.9 | | | 11.4 | |  |  |
|  |  | O (n=39) | 25.6 | 59.0 |  |  |  | 5.1 | | | 10.3 | |  |  |
|  |  | P (n=4) | 50.0 | 50.0 |  |  |  | 0.0 | | | 0.0 | |  |  |
|  | ROUND 3 | M (n=26) | 76.9 | 23.1 |  |  |  |  | | |  | |  |  |
|  |  | O (n=42) | 38.1 | 61.9 |  |  |  |  | | |  | |  |  |
| For how many minutes would you feel for contractions in low maternal and high foetal risk pregnancies during the active phase of labour? |  |  | 5 minutes | 10 minutes | Other | 5 minutes when not on medication stimulation and 10 minutes if on induction/augmentation* |  |  | | | | | YES (10 MINUTES) | |
|  | ROUND 1 | M (n=43) | 34.9 | 60.5 | 4.7 |  |  |  | | | | |  |  |
|  |  | O (n=47) | 25.5 | 72,3 | 2.1 |  |  |  |  |  |  |  |  |  |
|  |  | P (n=8) | 75.0 | 12.5 | 12.5 |  |  |  |  |  |  |  |  |  |
|  | ROUND 2 | M (n=35) | 11.4 | 60.0 | 2.9 | 25.7 |  |  |  |  |  |  |  |  |
|  |  | O (n=39) | 15.4 | 61.5 | 0.0 | 23.1 |  |  |  |  |  |  |  |  |
|  |  | P (n=4) | 50.0 | 25.0 | 0.0 | 25.0 |  |  |  |  |  |  |  |  |
|  | ROUND 3 | M (n=26) | 7.7 | 84.6 |  | 7.7 |  |  |  |  |  |  |  |  |
|  |  | O (n=42) | 11.9 | 76.2 |  | 11.9 |  |  |  |  |  |  |  |  |
| In case foetal heart beat is suboptimal, what is the minimal acceptable frequency of foetal heart monitoring in the first stage of active phase of labour, when only intermittent auscultation is available? |  |  | After every contraction | Every 5 minutes | Every 15 minutes | Every 30 minutes | Other |  | | | | | NO | |
|  | ROUND 1 | M (n=41) | 17.1 | 17.1 | 39 | 14.6 | 12.2 |  | | | | |  |  |
|  |  | O (n=47) | 27.7 | 19.1 | 31.9 | 8.5 | 12.8 |  |  |  |  |  |  |  |
|  |  | P (n=8) | 12.5 | 50.0 | 12.5 | 25.0 | 0.0 |  |  |  |  |  |  |  |
|  | ROUND 2 | M (n=35) | 54.3 | 14.3 | 22.9 | 8.6 | 0.0 |  |  |  |  |  |  |  |
|  |  | O (n=39) | 53.8 | 17.9 | 15.4 | 12.8 | 0.0 |  |  |  |  |  |  |  |
|  |  | P (n=4) | 50.0 | 25.0 | 25.0 | 0.0 | 0.0 |  |  |  |  |  |  |  |
|  | ROUND 3 | M (n=26) | 65.4 | 15.4 | 19.2 |  |  |  |  |  |  |  |  |  |
|  |  | O (n=42) | 78.6 | 14.3 | 7.1 |  |  |  |  |  |  |  |  |  |
| In case foetal heart beat is suboptimal, what is the minimal acceptable frequency of foetal heart monitoring in the second stage of active phase of labour, when only intermittent auscultation is available? |  |  | After every contraction | Every 5 minutes | Every 15 minutes | Every 30 minutes | Other |  | | | | | YES (AFTER EVERY CONTRACTION) | |
|  | ROUND 1 | M (n=41) | 65.9 | 14.6 | 9.8 | 4.9 | 4.9 |  | | | | |  |  |
|  |  | O (n=47) | 74.5 | 10.6 | 2.1 | 0.0 | 12.8 |  |  |  |  |  |  |  |
|  |  | P (n=8) | 62.5 | 12.5 | 25 | 0.0 | 0.0 |  |  |  |  |  |  |  |
|  | ROUND 2 | M (n=35) | 80.0 | 11.4 | 5.7 |  | 2.9 |  |  |  |  |  |  |  |
|  |  | O (n=39) | 79.5 | 7.7 | 2.6 |  | 10.3 |  |  |  |  |  |  |  |
|  |  | P (n=4) | 75.0 | 25.0 | 0.0 |  | 0.0 |  |  |  |  |  |  |  |
| In case foetal heart rate is abnormal within how many minutes should foetal heart be confirmed (before the decision for expedite delivery can be made)?* |  |  | 5 minutes | 10 minutes | 15 minutes | 30 minutes | 60 minutes | Other | | | 0 minutes* | | NO | |
|  | ROUND 2 | M (n=33) | 39.4 | 21.2 | 15.2 | 6.1 | 3.0 | 15.2 | | |  | |  |  |
|  |  | O (n=38) | 23.7 | 18.4 | 23.7 | 7.9 | 0.0 | 26.3 | | |  |  |  |  |
|  |  | P (n=4) | 60 | 20 | 0.0 | 0.0 | 0.0 | 20 | | |  |  |  |  |
|  | ROUND 3 | M (n=26) | 69.2 | 11.5 | 3.8 |  | |  | | | 15.4 | |  |  |
|  |  | O (n=42) | 54.8 | 11.9 | 14.3 |  |  |  | | | 19.0 | |  |  |
| Legend: M= Midwives, O=Obstetricians, P = Paediatricians; The paediatric was deemed too small and hence consensus in round 2 and 3 was based on midwife and obstetrician groups.  *New option/question | | | | | | | | | | | | | | |
