# Supplementary material for: Delphi consensus statement on intrapartum fetal monitoring in low‐resource settings
Source: Int J Gynaecol Obstet. 2018 Dec 24;146(1):8–16. doi: 10.1002/ijgo.12724 (PMC7379246; doi:10.1002/ijgo.12724)
Supplement: Supplementary file 3 — Table S3. Feedback from participants of Delphi rounds and/or consultation meeting. [file IJGO-146-8-s003.docx]

| **Table S3 Feedback from participants of Delphi rounds and/or consultation meeting** | |
| --- | --- |
| **Theme** | **Details** |
| Definitions | -clarification on e.g. low-resource setting, admission test, low/high risk, suboptimal/abnormal FHR. |
| Additional outcomes | - E.g. what should constitute admission tests and adjunctive tests; frequency of FHR and contraction monitoring  -Emphasis on structured approach , technique and interpretation of assessments including timing FHR with contractions and simultaneous palpation of maternal pulse.  -Maternal positional change and reducing contractions as the most relevant procedures of intrauterine resuscitation.  -discouragement of routine rupture of membranes to check for meconium, i.e. check only when membranes are already ruptured.  -Encourage use of vacuum/forceps in second stage of labour |
| Achievability | -Recommended frequency of monitoring likely not achievable and hence not respectful to the overwhelmed healthcare professionals.  -Context should be stratified according to availability of resources to inform the development of context-specific, achievable guidance.  -Advantages and disadvantages of monitoring devices including:  -Prerequisite for use of CTG: availability of equipment and consumables, trained personnel for interpretation and access to theatre; able to detect subtle changes in FHR e.g. variability  -Invasive tests: infection  -Pinard: comparable to hand-held Doppler but requires high skills and quiet setting.  -Hand-held Doppler: costly but user- and mother-friendly |
| Evidence | -Unavailability of evidence as an inherent limitation on expert-opinion  -The need for further research:  -Research priorities: Impact of admission tests, methods of IA, stimulation tests, maternal perception of foetal movement, intrauterine resuscitation and decision-delivery time on perinatal outcomes.  - implementation research, adaptive clinical trials and qualitative approaches on the experiences of health professionals and patients in order to tackle research questions on intrapartum monitoring in low resource settings.  - investment in innovating appropriate technologies which are affordable and functioning under all conditions |
| CTG = Cardiotocography, FHR = Foetal heart rate monitoring, IA = Intermittent auscultation | |
