# Supplementary material for: Delphi consensus statement on intrapartum fetal monitoring in low‐resource settings
Source: Int J Gynaecol Obstet. 2018 Dec 24;146(1):8–16. doi: 10.1002/ijgo.12724 (PMC7379246; doi:10.1002/ijgo.12724)
Supplement: Supplementary file 4 — Table S4. Attrition analysis. [file IJGO-146-8-s004.docx]

| Table S4 Attrition analysis | | | | | | |  |
| --- | --- | --- | --- | --- | --- | --- | --- |
|  | **Round 1 results of non-respondent to round 2** | **Round 1 results of non-respondent to round 3** | **Round 1 results of respondents to round 3** | **Round 2 results of non-respondents**  **to round 3** | **Round 2 results of respondents to round 3** | **Round 3 results of respondents to round 3** |  |
| Would you be in favour of an admission test to assess foetal well-being? | 5(3-5) | 5(4-5) | 5(4-5) |  |  | 5(5-5) |  |
| If you are to use an admission test for pregnant woman in labour in a low resource setting, how do you rate EACH of the following for the assessment of foetal wellbeing on admission to the labour ward in a low resource setting? | | | | | | | |
| Cardiotocogram (CTG) | 4(2-4) | 4(2-5) | 3(2-4) | 4(2-4) | 2(1-4) | 2(1-3) |  |
| Foetal heart rate auscultation by Pinard/DeLee stethoscope | 4(4-5) | 5(4-5) | 4(4-5) | 5(2-5) | 5(4-5) |  |  |
| Foetal heart rate auscultation by hand-held Doppler | 4(3-5) | 4(3-5) | 5(4-5) |  |  |  |  |
| Amniotic Fluid Index/assessment | 3(2-3) | 3(2-5) | 3(2-4) | 2(1-3) | 2(1-3) | 1(1-2) |  |
| Foetal Acoustic Stimulation Test | 2(1-3) | 3(2-4) | 3(1-3) | 3(2-4) | 2(1-3) | 1(1-2) |  |
| Foetal Movement Assessment by maternal perception | 4(3-5) | 4(4-5) | 4(3-5) | 4(4-5) | 4(4-5) |  |  |
| Foetal Movement Assessment by ultrasound detection | 2(1-4) | 3(2-4) | 2(1-3) | 3(2-4) | 2(1-3) | 1(1-2) |  |
| Rapid Biophysical profile (ultrasound detection of foetal movement and Amniotic Fluid Index) | 2(1-3) | 2(2-4) | 2(1-3) | 2(1-3) | 2(1-3) | 1(1-2) |  |
| Umbilical Artery Doppler assessment | 2(1-3) | 2(2-4) | 1(1-3) | 2(1-2) | 1(1-2) | 1(1-2) |  |
| Foetal Pulse Oximetry | 2(1-3) | 2(1-4) | 1(1-3) | 1(1-2) | 1(1-2) | 1(1-2) |  |
| Assessment of meconium stained liquor | 4(4-5) | 4(4-5) | 4(3-5) | 4(2-5) | 4(4-5) | 5(4-5) |  |
| Assessment of gestational age | 4.5(4-5) | 4(4-5) | 4(4-5) |  | | |  |
| Assessment of fundal height | 4(4-5) | 5(4-5) | 4(4-5) |  |  |  |  |
| Assessment of maternal blood loss | 4(4-5) | 5(4-5) | 4(3-5) |  |  |  |  |
| Foetal Scalp Stimulation Test{NEW OPTION} |  |  |  | 3(1-3) | 3(1-3) | 2(1-2) |  |
| What would be the appropriate method for foetal heart rate monitoring  for low maternal and low foetal risk pregnancies in the first stage of active phase of labour? | | | | | | | |
| Cardiotocogram (CTG) - Non-invasive (abdominal) | 2(1-4) | 4(2-5) | 2(1-3) | 2(1-4) | 2(1-3) | 2(1-3) |  |
| Cardiotocogram (CTG) - Invasive (foetal scalp electrodes) | 1(1-2) | 2(1-3) | 1(1-2) |  | | |  |
| Intermittent Auscultation – Pinard/DeLee stethoscope | 4(4-5) | 5(4-5) | 4(4-5) | 4(3-5) | 5(4-5) |  |  |
| Intermittent Auscultation - Hand-held Doppler | 4(3-5) | 4(3-5) | 5(4-5) |  | | |  |
| In a low resource setting, what would be your method for foetal heart rate monitoring for low maternal and low foetal in the second stage of active phase of labour? | | | | | | | |
| Cardiotocogram (CTG) - Non-invasive | 3(1-5) | 4(3-5) | 2(1-4) | 2(1-4) | 2(1-3) | 2(1-2) |  |
| Cardiotocogram(CTG) - Invasive | 1(1-2) | 2(1-2) | 1(1-2) |  | | |  |
| Intermittent Auscultation -Pinard/DeLee stethoscope | 4(3-5) | 4(3-5) | 4(4-5) | 4(3-5) | 4(4-5) |  |  |
| Intermittent Auscultation - Hand-held Doppler | 4(4-5) | 4(4-5) | 5(4-5) | 4(3-5) | 5(4-5) |  |  |
| In a low resource setting, what would be your method for foetal monitoring in low maternal, high foetal risk pregnancies in the first stage of active phase of labour? | | | | | | | |
| Cardiotocogram (CTG) - Non-invasive | 5(4-5) | 4(4-5) | 4(3-5) | 4(4-5) | 4(4-5) | 4(3-5) |  |
| Cardiotocogram(CTG) - Invasive | 2(1**-**3) | 3(1-3) | 2(1-3) | 2(1-3) | 2(1-3) | 2(1-3) |  |
| Intermittent Auscultation -Pinard/DeLee stethoscope | 4(3-4) | 4(3-4) | 4(3-5) | 4(2-5) | 4(3-5) |  |  |
| Intermittent Auscultation - Hand-held Doppler | 4(4-5) | 4(3-5) | 4(4-5) | 4(3-5) | 5(4-5) |  |  |
| In a low resource setting, what would be your method for foetal monitoring in low maternal, high foetal risk pregnancies in the second stage of active phase of labour? | | | | | | | |
| Cardiotocogram (CTG) - Non-invasive | 5(4-5) | 4(3-5) | 4(3-5) | 5(4-5) | 4(3-5) | 4(3-5) |  |
| Cardiotocogram(CTG) - Invasive | 2(1-5) | 3(2-4) | 2(1-4) | 2(1-3) | 2(1-3) | 2(1-3) |  |
| Intermittent Auscultation – Pinard/DeLee stethoscope | 4(2-4) | 4(3-5) | 4(3-5) | 5(2-5) | 4(3-5) |  |  |
| Intermittent Auscultation - Hand-held Doppler | 4(4-5) | 4(4-5) | 4(4-5) |  | | |  |
| When foetal heart rate is normal, which supplemental test would you like for monitoring foetal well-being? | | | | | | | |
| Foetal Scalp Sampling | 1(1-2) | 2(1-2) | 1(1-2) |  | | |  |
| Foetal Pulse Oximetry | 1(1-2) | 2(1-2) | 1(1-2) |  | | |  |
| Foetal Scalp Stimulation Test | 1(1-2) | 1(1-3) | 1(1-2) |  | | |  |
| Assessment of liquor for meconium | 4(3-4) | 5(4-5) | 4(2-5) | 5(4-5) | 4(3-5) | 4(4-5) |  |
| Assessment of foetal movements by maternal perception | 4(3-5) | 4(4-5) | 4(2-4) | 5(4-5) | 4(3-5) | 4(3-5) |  |
| Assessment of foetal movement by ultrasound detection | 2(1-3) | 3(1-3) | 2(1-2) | 2(1-4) | 2(1-2) | 1(1-2) |  |
| Foetal Acoustic Stimulation Test | 2(1-2) | 2(1-3) | 2(1-3) | 2(1-4) | 1(1-2) | 1(1-2) |  |
| None: No additional test, continue monitoring | 4(3-4) | 4(2-5) | 4(3-5) | 4(2-5) | 4(4-5) |  |  |
| Monitor maternal wellbeing {NEW OPTION} |  |  |  | 4(4-5) | 5(4-5) |  |  |
| In case foetal heart beat is suboptimal, what adjunctive test(s) would you do to confirm foetal well-being in the first stage of active phase of labour? | | | | | | | |
| Foetal Scalp Sampling | 3(2-5) | 2(1-4) | 3(2-4) | 2(1-4) | 3(2-4) | 2(1-3) |  |
| Foetal Pulse Oximetry24 | 3(1-4) | 2(1-3) | 2(1-3) | 2(1-2) | 2(1-3) | 2(1-2) |  |
| Foetal Scalp Stimulation Test | 3(1-3) | 2(2-4) | 3(2-4) | 3(2-4) | 3(2-4) | 3(2-4) |  |
| Assessment of liquor for meconium | 4(4-4) | 4(4-5) | 4(4-5) |  | | |  |
| Assessment of foetal movements by maternal perception | 4(3-5) | 4(2-5) | 3(2-4) | 3(2-5) | 4(2-5) | 4(2-4) |  |
| Assessment of foetal movement by ultrasound detection | 3(2-4) | 4(2-4) | 2(2-3) | 3(2-4) | 2(1-3) | 2(1-3) |  |
| Foetal Acoustic Stimulation Test | 3(1-3) | 3(2-4) | 2(1-4) | 3(2-4) | 2(1-3) | 2(1-2) |  |
| None: No additional test, continue monitoring | 1(1-2) | 2(1-4) | 2(1-4) | 2(2-3) | 2(1-3) | 2(1-3) |  |
| None: No additional test, immediate delivery | 2(1-2) | 2(1-4) | 2(1-3) | 2(2-4) | 2(1-3) | 2(1-2) |  |
| Intrauterine resuscitation {NEW OPTION} |  |  |  | 4(4-5) | 4(4-5) |  |  |
| Biophysical Profile ({NEW OPTION} |  |  |  | 2(1-3) | 2(1-3) | 2(1-3) |  |
| In case foetal heart beat is suboptimal, what adjunctive test(s) would you do to confirm foetal well-being in the second stage of active phase of labour? | | | | | | | |
| Foetal Scalp Sampling | 2(1-5) | 3(2-4) | 2(1-4) | 2(2-4) | 2(1-3) | 2(1-2) |  |
| Foetal Pulse Oximetry | 2(1-4) | 2(1-3) | 2(1-3) | 2(2-3) | 2(1-2) | 1(1-2) |  |
| Foetal Scalp Stimulation Test | 1(1-3) | 2(2-4) | 3(1-4) | 2(2-4) | 2(2-4) | 2(1-3) |  |
| Assessment of liquor for meconium | 4(3-4) | 4(4-5) | 4(3-5) | 5(4-5) | 4(3-5) | 4(4-5) |  |
| Assessment of foetal movements by maternal perception | 4(2-5) | 4(2-5) | 3(2-4) | 3(2-4.25) | 3(2-4) | 2(1-4) |  |
| Assessment of foetal movement by ultrasound detection31 | 2(1-3) | 3.5(2-4) | 2(1-3) | 2(2-3) | 2(1-2) | 1(1-2) |  |
| Foetal Acoustic Stimulation Test | 2(1-3) | 4(2-4) | 2(1-3) | 2(1-3) | 2(1-2) | 2(1-2) |  |
| None: No additional test, continue monitoring | 1(1-3) | 2(1-3) | 2(2-4) | 2(1-3) | 2(1-3) | 2(1-3) |  |
| None: No additional test, immediate delivery | 2(1-5) | 3(1-4) | 3(2-5) | 4(2-4) | 4(2-5) | 2(2-3) |  |
| Intrauterine resuscitation {NEW OPTION} |  |  |  | 4(4-5) | 4(4-5) | 5(4-5) |  |
| Biophysical Profile ({NEW OPTION} |  |  |  | 3(1-3) | 2(1-3) | 1(1-2) |  |
| In case foetal heart beat is abnormal, what adjunctive test(s) would you do to confirm foetal well-being in the first stage of active phase of labour? | | | | | | | |
| Foetal Scalp Sampling | 1.5(1-4) | 3(1-4) | 3(2-4) | 2(1.5-4) | 3(2-4) | 3(2-4) |  |
| Foetal Pulse Oximetry | 2(1-3) | 1.5(1-4) | 2(1-3.25) | 2(1.5-2) | 2(1-3) | 2(1-2) |  |
| Foetal Scalp Stimulation Test | 2(1-3) | 3.5(1-4) | 3(2-4) | 2(1.5-4) | 2(1-4) | 3(1-4) |  |
| Assessment of liquor for meconium | 4(2-5) | 4(3-4) | 4(3-5) | 5(4-5) | 4(4-5) |  |  |
| Assessment of foetal movements by maternal perception | 3(2-4) | 4(1-5) | 3(2-4) | 2(2-4) | 3(2-4) | 2(1-4) |  |
| Assessment of foetal movement by ultrasound detection | 3(1-4) | 4(1-4) | 2(1-4) | 2(2-4) | 2(1-3) | 2(1-2) |  |
| Foetal Acoustic Stimulation Test | 2(1-3) | 3(2-4) | 2(1-3) | 2(1-3) | 2(1-3) | 2(1-2) |  |
| None: No additional test, continue monitoring | 1(1-3) | 1(1-3) | 2(1-3) | 2(1-3) | 2(1-2) | 2(1-2) |  |
| None: No additional test, immediate delivery | 3(1-5) | 4(1-5) | 4(2-4) | 3(2-4.5) | 4(2-4) | 4(2-5) |  |
| Intrauterine resuscitation {NEW OPTION} |  |  |  | 4(4-5) | 4(4-5) |  |  |
| Biophysical Profile {NEW OPTION} |  |  |  | 2(1-3) | 2(1-4) | 1(1-2) |  |
| In case foetal heart beat is abnormal, what adjunctive test(s) would you do to confirm foetal well-being in the second stage of active phase of labour? | | | | | | | |
| Foetal Scalp Sampling | 1(1-4) | 4(2-4) | 2(1-4) | 1(1-3) | 2(1-3) | 2(1-3) |  |
| Foetal Pulse Oximetry | 2(1-4) | 2(1-4) | 2(1-4) | 1(1-2) | 2(1-3) | 1(1-2) |  |
| Foetal Scalp Stimulation Test | 2(1-3) | 4(2-4) | 3(2-4) | 2(1-3) | 2(1-3) | 2(1-3) |  |
| Assessment of liquor for meconium | 4(1-4) | 4(4-5) | 4(2-4) | 5(4-5) | 4(4-5) | 4(4-5) |  |
| Assessment of foetal movements by maternal perception | 3(2-4) | 4(2-5) | 2(1-3) | 3(2-4) | 2(1-4) | 2(1-4) |  |
| Assessment of foetal movement by ultrasound detection | 2(1-4) | 3(1-4) | 2(1-3) | 2(2-3) | 1(1-2) | 1(1-2) |  |
| Foetal Acoustic Stimulation Test | 2(1-3) | 3(2-4) | 2(1-3) | 2(1-3) | 1(1-2) |  |  |
| None: No additional test, continue monitoring | 1.5(1-3) | 1(1-2) | 2(1-3) | 2(1-3) | 2(1-3) | 2(1-2) |  |
| None: No additional test, immediate delivery | 5(2-5) | 2(1-4) | 4(3-5) | 5(3-5) | 4(4-5) | 4(4-5) |  |
| Intrauterine resuscitation ({NEW OPTION} |  |  |  | 5(4-5) | 4(4-5) | 5(4-5) |  |
| Biophysical Profile ({NEW OPTION} |  |  |  | 2(2-3) | 2(1-2) | 1(1-2) |  |
| Legend: Numbers are in median (range) | | | | | | |  |
