# Supplementary material for: Delphi consensus statement on intrapartum fetal monitoring in low‐resource settings
Source: Int J Gynaecol Obstet. 2018 Dec 24;146(1):8–16. doi: 10.1002/ijgo.12724 (PMC7379246; doi:10.1002/ijgo.12724)
Supplement: Supplementary file 5 — Table S5. References for guidelines listed in Table 1. [file IJGO-146-8-s005.docx]

**Table S5** References for guidelines listed in Table 1.

| **No.** | **Reference** |
| --- | --- |
| 1 | Lewis D, Downe S. FIGO consensus guidelines on intrapartum fetal monitoring: Intermittent auscultation. Int J Gynecol Obstet 2015;131:9–12 |
| 2 | WHO, Managing Complications in Pregnancy and Childbirth: A guide for midwives and doctors, 2000 |
| 3 | National Institute for Health and Clinical Excellence. Intrapartum care for health healthy women and babies. NICE 2014:1–58. |
| 4 | The Royal Australian and New Zealand College of Obstetricians and Gynaecologists. Intrapartum Fetal Surveillance. 2014. |
| 5 | The American College of Nurse-midwives. Intermittent Auscultation for Intrapartum Fetal Heart Rate Surveillance (replaces ACNM Clinical Bulletin #9, March 2007). J Midwifery Women’s Heal 2010;55:397–403. |
| 6 | American College of Obstetrics and Gynecology. Intrapartum Fetal Heart Rate Monitoring: Nomenclature, Interpretation, and General Management Principles. Obstet Gynecol 2009;114:192–202. |
| 7 | The Society of Obstetricians and Gynaecologists of Canada (SOGC). Fetal health surveillance: antepartum and intrapartum consensus guideline. J Obstet Gynaecol Can 2007;29:S3–56. |
| 8 | The Royal College of Obstetrics and Gynaecology. The use of electronic monitoring: The use and interpretation of cardiotocography in intrapartum fetal surveillance. Clinical Guideline number 8. 2001. |
